# Target of rapamycin signaling in pea embryos is dependent on glutamine but detached from seed storage protein biosynthesis

**DOI:** 10.1111/nph.70622

**Published:** 2025-10-15

**Authors:** Brendan M. O'Leary, Suvi Honkanen, Vinti Kumari, Christoph Rampitsch, Eiji Nambara, A. Harvey Millar

**Affiliations:** ^1^ Saskatoon Research and Development Centre, Agriculture and Agri‐Food Canada Saskatoon S7N 0X2 Canada; ^2^ Department of Biology University of Saskatchewan Saskatoon S7N 0X2 Canada; ^3^ Global Institute for Food Security University of Saskatchewan Saskatoon S7N 4L8 Canada; ^4^ Morden Research and Development Centre, Agriculture and Agri‐Food Canada Morden R6M 1Y5 Canada; ^5^ Department of Cell and Systems Biology University of Toronto Toronto M5S 3G5 Canada; ^6^ Centre for Plant Energy Biology and School of Molecular Sciences University of Western Australia Perth WA 6009 Australia

**Keywords:** amino acid metabolism, glutamine, phosphoproteomics, *Pisum sativum*, protein synthesis, seed development, seed storage proteins, TOR signaling

## Abstract

- Target of rapamycin (TOR) kinase is the hub of a eukaryotic master signaling network that integrates nutritional and hormonal signals into cellular activities. Most studies on TOR in plants have focused on seedlings, where TOR is most responsive to light and sucrose. Here, we observed differences in nutrient regulation of TOR across plant tissues.
- Biochemical analyses highlighted the predominance of Gln‐TOR signaling in mature Arabidopsis leaves and developing pea seeds, and its integration with hormone signaling and amino acid metabolism. Phosphoproteomic and transcriptomic analysis of developing pea seeds identified established and novel components of TOR signaling, which were enriched for proteins/genes regulating gene expression and autophagy.
- Unexpectedly, Gln‐TOR signaling in pea embryos inhibited or delayed growth and protein accumulation during seed filling. A developmental profile was evident wherein high TOR activity and Gln levels during pea cotyledon cellularization reduced sharply as embryos progressed to seed filling. We observed strong interactions between TOR and abscisic acid (ABA) signaling such that TOR‐inhibited embryos were hypersensitive to ABA‐induced protein accumulation.
- We propose that legume seed storage protein biosynthesis displays atypical regulatory properties because it occurs in the face of increasing desiccation stress and is promoted by ABA signaling rather than TOR signaling.

## Introduction

In all eukaryotes, protein complexes containing target of rapamycin (TOR) kinase are central coordinators of cell growth and proliferation (Liu & Sabatini, 2020). While mammals contain two TOR complexes, TORC1 and TORC2, plants appear to only contain the TORC1 complex, which consists of TOR, LST8 and RAPTOR subunits (Shi *et al*., 2018). TORC1 complexes play a major role in sensing energy, nutrient and hormone levels and regulating cellular activity accordingly (Valvezan & Manning, 2019). When nutrients are available, TORC1 promotes growth and anabolic pathways such as the cell cycle and protein synthesis, but when nutrients become scarce, diminished TORC1 activity leads to growth suppression alongside upregulation of catabolic pathways and autophagy to obtain nutrients by cellular recycling (Liu & Sabatini, 2020; Meng *et al*., 2022).

The upstream and downstream components of the TORC1 signaling pathway have diversified throughout eukaryotes, reflecting the differing nutritional, developmental and metabolic lifestyles across kingdoms (Wolfson & Sabatini, 2017; Shi *et al*., 2018; Lutt & Brunkard, 2022). Compared to animals and yeast, the upstream components of TORC1 signaling in plants are poorly characterized and important nutritional, hormonal or environmental inputs likely remain uncharacterized. It is also unclear if TOR nutrient regulators will be consistent across plant tissues. We focus here on amino acids, which are critical upstream activators of TORC1 activity in animals and yeast. In animal cells, specific amino acid levels (e.g. Leu, Arg and S‐Ado‐Met) are detected by several mechanisms that converge upon recruitment and activation of TORC1 by Rag and Rheb GTPases at the lysosomal membrane (Duran *et al*., 2012; Wolfson & Sabatini, 2017). Multiple studies in yeast and animals have also reported TOR activation by Gln levels in a Rag GTPase‐independent mechanism (Stracka *et al*., 2014; Jewell *et al*., 2015; Meng *et al*., 2020; Fernandes *et al*., 2024). By contrast, amino acids were only recently demonstrated to be metabolite activators of TORC1 in select plant tissues (Cao *et al*., 2019; O'Leary *et al*., 2020; Liu *et al*., 2021; Lutt & Brunkard, 2022; Li *et al*., 2024). However, the amino acid‐sensing components established in animals and yeast are mostly not conserved in green algae or plants, which lack orthologues to Rheb and Rag GTPases and the amino acid‐sensing proteins Sestrins, CASTORs, SAMTOR and SLC38A9 (Liu & Sabatini, 2020). One exception could be yeast protein Pib2 and its plant homolog FYVE/FREE1, both of which appear to activate TORC1 in a Gln‐dependent manner (Tanigawa *et al*., 2024). Given the fundamentally different metabolic lifestyles in autotrophic plants vs heterotrophic fungi and animals, it is unclear which functionalities of amino acid TOR signaling are conserved vs newly recruited across the different eukaryotic lineages.

TOR's relationship to plant N‐metabolism has been the focus of recent studies in light of the importance of N‐uptake, assimilation and protein synthesis to both natural ecosystems and agricultural crop production systems. Several aspects of TORC1 signaling create regulatory linkages between sensing amino acid levels and directing their use in the cell. For example, the stimulation of protein translation by TORC1 activation creates a direct need for amino acids (Schepetilnikov *et al*., 2017; Scarpin *et al*., 2020). TORC1's promotion of ribosome biosynthesis and stimulation of the cell cycle necessitate the biosynthesis of N‐rich nucleotides, which require amino acid precursors (Busche *et al*., 2021). Conversely, TORC1 inhibition in plants strongly increases cellular amino acid levels, particularly Gln and the branched chain amino acids (Moreau *et al*., 2012; Ren *et al*., 2012; Caldana *et al*., 2013; Mubeen *et al*., 2018; Ingargiola *et al*., 2023). This replenishment of amino acids is likely partly due to derepression of macro‐autophagy and thus vacuolar protein degradation (Caldana *et al*., 2013). However, in green algae, the increase in amino acid levels upon TOR inhibition was mostly attributable to increased *de novo* amino acid biosynthesis, with a relatively minor contribution from increased protein degradation (Mubeen *et al*., 2018). For each of these effects, the role of TOR in sensing and regulating amino acid levels is homeostatic, as it acts to maintain balance in nutrient supply and demand (Valvezan & Manning, 2019).

The effects of TOR regulation on plant inorganic nitrogen uptake and assimilation are less clear. Chemical inhibition or genetic disruption of TORC1 reduced ammonium uptake and measurable glutamine synthetase activity in Arabidopsis seedlings (Ingargiola *et al*., 2023). However, these TOR‐compromised seedlings contained greater quantities of ammonium and Gln. Similarly, TOR inhibition in rice strongly repressed the translation of the ammonium transporter AMT1 (Li *et al*., 2024). Conversely, in *Chlamydomonas reinhardtii*, TOR inhibition stimulates the uptake and assimilation of ^15^NH_4_^+^ into amino acids (Mubeen *et al*., 2018). The above observations in plants could conceptually exacerbate a shortage of cellular amino acids because of a reduction in N uptake and assimilation. However, TOR inhibition also increases protein turnover, enhancing cellular amino acid levels and this may feedback to inhibit *de novo* amino acid biosynthesis. More information is required to clarify the TOR‐nitrogen relationship across the plant kingdom.

Across eukaryotes, maximal TORC1 activation requires multiple inputs to be present. Besides amino acids, TORC1 activity in plants is highly responsive to glucose levels, respiratory activity, several hormone signaling pathways and SnRK1 kinase activity (Liu *et al*., 2025). The discovery of a growing number of upstream metabolites and protein regulators of plant TORC1 enhances the likelihood that TORC1 regulatory properties differ between tissues to suit their distinct metabolic and developmental requirements. However, most studies on plant TORC1 signaling so far have occurred in Arabidopsis seedlings, with a focus on root and shoot meristems. In this study, we contrast the nutritional regulation of TOR across mature Arabidopsis leaves, seedling roots and shoots and developing pea embryos. We identify Gln as the sole metabolite activator of TOR in developing pea seeds and report regulatory differences between tissues, including TORC1's effect on protein synthesis and the light dependence of TORC1 activation. Legume seed Gln‐TOR signaling is hypothesized to antagonize seed ABA signaling and together, these two pathways help dictate the timing of the cellular transition to storage protein accumulation. This regulation of seed development by Gln‐TOR signaling has important implications toward maximizing yield in agricultural crops such as pulses.

## Materials and Methods

### Plant material and growth conditions

Arabidopsis (*Arabidopsis thaliana* (L.) Heynh.) accession Col‐0 (N6000) was the wild‐type. For immunoblotting of S6K, the *35S‐S6K1‐HA* line (At3g08730) was used (Van Leene *et al*., 2019). Seeds were sown into a 3 : 1 : 1 mix of potting soil, perlite and vermiculite, supplemented with slow‐release fertilizer. Plants were grown under a short‐day photoperiod of 8 h of light and 16 h of dark with a photon flux of 120 μmol m^−2^ s^−1^, 75% relative humidity and day : night temperatures of 22°C to 17°C. Leaf discs (7 mm diameter) were harvested from the mature leaves of 7‐ to 9‐wk‐old plants. Arabidopsis seedlings were grown on 0.7% Phytagel plates (Sigma) containing half‐strength, nitrogen‐free, Murashige & Skoog (MS) media (MSP07; Plant Cell Labs, Smithfield, UT, USA), supplemented with 5 mM KNO_3_ and 5 mM MES, pH 5.6. Plates were maintained under 16 h light with a photon flux of 120 μmol m^−2^ s^−1^ and seedlings were selected for experiments at between one and 2 wk old.

Pea (*Pisum sativum* L.) cultivar Cameor was grown in a glasshouse under natural light supplemented with 16 h of full‐spectrum LED lighting. Plants were grown in 4 l round pots filled with high porosity soil (Promix HP) supplemented with 0.2 g l^−1^ FeEDTA. Soil was inoculated with 1.25 g l^−1^ of Primo GX2 pulse granular inoculant (Verdesian, Cary, NC, USA) and watered on alternating days. Temperature was maintained between 22°C and 18°C.

### Tissue incubations

Chemical treatments of leaf discs were conducted by floating leaf discs in the dark on 600 μl buffer containing 50 mM HEPES, 10 mM MES, pH 6.6 and 200 μM CaCl_2_ plus additional chemicals as indicated, within open tubes. Leaf discs were harvested for incubation between 3 and 4 h into the light or dark period. Where specified, light treatments involved exposure to 120 μmol m^−2^ s^−1^ light.

For incubations of pea embryos, seeds were removed from freshly harvested pods, sterilized in 30% bleach for 5 min and placed in distilled water. Entire embryos were then dissected away from seed coats under sterile conditions and weighed. Embryos were then incubated at room temperature in 1.6 ml of sterile media in 12‐well plates on an orbital shaker. The media composition, based on previous studies (Millerd *et al*., 1975; Thompson *et al*., 1977), was 3.23 g l^−1^ nitrogen‐free MS media (MSP07; Caisson Labs), 5 mM MES pH 5.7, 5% (w/v) sucrose and 62.5 mM Gln, with modifications as indicated. Due to thermal instability, it is necessary to add glutamine after autoclaving the media, followed by filter sterilization (Thompson *et al*., 1977). Unless specified, embryos were exposed to 16 h of light (100 μmol m^−2^ s^−1^) and 8 h of darkness.

### Immunoblotting

Samples consisting of individual embryos were extracted in 100 mM HEPES, pH 7.5, 25 mM glycerol‐2‐phosphate, 5 mM NaF, 2.5 mM sodium pyrophosphate, 25 mM β‐phosphoglycerol, 0.1% (v/v) Triton X‐100, 2 mM PMSF and 1% (v/v) ProteCEASE protease inhibitor cocktail (G‐Bioscience, St Louis, MI, USA) combined 1 : 1 with SDS sample buffer. For immunoblotting against S6K, 12% acrylamide SDS‐PAGE gels were supplemented with 6 M urea to facilitate S6K resolution (Urea‐SDS‐PAGE). For immunoblotting against RPS6, normal SDS‐PAGE gels were used. The following antibodies were used: anti‐HA antibody (catalogue No. 66006‐2‐lg; Proteintech, Rosemont, IL, USA) at 1 : 20 000 dilution; anti‐S6K (catalogue No. AS12 1855; Agrisera, Vännäs, Sweden) at 1 : 1000 dilution; anti‐S6K‐phosphoT449 antibody (catalogue No. 207399, lot No. GR243231‐22; Abcam, Cambridge, UK) at 1 : 1000; anti‐RPS6 (catalogue No. AS19 4292; Agrisera) at 1 : 1000; anti‐RPS6‐phosphoS237 (catalogue No. AS19 4291; Agrisera) at 1 : 1000 or anti‐RPS6‐phosphoS240 (catalogue No. AS19 4302; Agrisera) at 1 : 1000. Following exposure, immunoreactive bands were quantified using ImageLab software (BioRad).

### Metabolite analysis

For leaf metabolomics, 6‐h leaf disc incubations were performed as above in media containing 10 mM ^15^NH_4_^15^NO_3_ beginning at 2 h into the night period for both light and dark‐treated samples. Samples consisting of two leaf discs were rinsed briefly in distilled water, patted dry on a paper towel, then snap‐frozen in liquid N_2_ and powdered using a bead mill. Gas chromatography–mass spectrometry metabolite analysis was performed as described previously (O'Leary *et al*., 2017), using ribitol as an internal standard. Nitrogen isotope abundance distributions for amino acids were calculated using IsoCor (Millard *et al*., 2019). The most abundant *m*/*z* ion series representing a TMS‐labeled amino acid with the loss of its α‐carboxylic acid group was selected as the typical quantified amino acid fragment (see Fig. 3, see later).

Pea embryo sucrose and starch measurements were performed by pulverizing single pea embryos in a bead mill, followed by metabolite extraction in 1 ml of 80% methanol for 15 min at 65°C and 160 ***g*** on a thermomixer. Following centrifugation, sucrose was assayed by diluting 60 μl aliquots of supernatant into 180 μl of 40 mM sodium acetate, pH 4.5, containing 125 U of invertase and incubating for 30 min at 50°C and 800 rpm. Glucose units were then measured using the d‐glucose assay kit (GOPOD format; Megazyme, Bray, Ireland). Starch was measured based on the method of (Smith & Zeeman, 2006) using a starch assay kit (Megazyme). Glutamine was measured by treating metabolite extracts with glutaminase (NZY Tech, Lisbon, Portugal), followed by coupling the resultant ammonia production to the oxidation of NADH using α‐ketoglutarate and glutamate dehydrogenase. Ammonia concentration was determined using a standard curve of ammonia and the reaction progression was monitored at 340 nm in a microplate reader.

### Protein synthesis quantification

Leaf protein synthesis rates were measured according to the method of O'Leary *et al*. (2017). Briefly, leaf discs were incubated with 0.1 mCi of uniformly labeled [^14^C]Leu (300 mCi mmol^−1^; Perkin Elmer, Waltham, MA, USA) in the presence or absence of 2 μM AZD and 10 mM Gln as indicated. Directly afterward, individual leaf discs were rinsed, frozen in N_2_, ground in a bead mill and proteins and amino acids were extracted in 0.1 M NaOH. Protein was then precipitated overnight with 5% TCA at 4°C. Following centrifugation, the pellet was resolubilized in 0.1 M NaOH containing 0.5% SDS. The TCA pellet and supernatant fractions were measured by scintillation counting to determine protein incorporated and free [^14^C]Leu.

For pea embryo protein synthesis measurements, individual embryos were weighed before and after 24‐h incubations, which were conducted as above. Embryos were then immediately pulverized in a bead mill, followed by protein extraction in 2% SDS and 50 mM Tris, pH 8.5. Protein quantification was performed using a BCA protein assay kit (Thermo Scientific, Waltham, MA, USA).

### Protein MS/MS

Phosphopeptide preparation was based on the method of Humphrey *et al*. (2018). Pea embryos, *c*. 100 mg in size, were incubated as indicated and then pulverized in a bead mill. Protein was extracted in 1000 μl of 4% sodium deoxycholate, 50 mM Tris pH 8.5, 2 mM EDTA, 0.5 mM sodium vanadate, 5 mM sodium pyrophosphate, 5 mM β‐glycerophosphate, 20 mM sodium fluoride and 0.1% Triton X‐100 at 95°C for 5 min. Samples were sonicated for 10 min with continuous 30 s on 30 s off cycles at max intensity in a Bioruptor (Diagenode, Liège, Belgium). Samples were then mixed with 4 ml of acetone and incubated overnight at −20°C, followed by centrifugation. Pellets were washed once with acetone, centrifuged and the pellet solubilized in 100 μl 4% SDC in 100 mM Tris, pH 8. Protein amount was measured using a BCA assay kit (Pierce, Appleton, WI, USA). One thousand micrograms of protein for each sample was then incubated with 10 mM DTT at 56°C for 45 min, followed by incubation with 55 mM iodoacetamide for 30 min in the dark and overnight digestion with 20 μg of a Trypsin/Lys C mixture while incubating at 37°C. Phosphopeptide enrichment was performed using 3 mg Titanosphere cartridges (GL Sciences, Eindhoven, the Netherlands) following the manufacturer's instructions. The final eluant was desalted using a mono‐spin C18 cartridge (GL Biosciences) following the manufacturer's nd dried down using a vacuum concentrator.

Mass spectrometry was performed on a Q‐Exactive LC‐MS system (ThermoFisher Scientific). Samples were dissolved in 5 μl of 1% (v/v) formic acid in 2% (v/v) acetonitrile (ACN) and injected into a nanospray column (18 cm fused silica column, 75 μm ID, packed with Luna C18, 5 μm beads, 100 Å pores) coupled directly to the mass spectrometer via a nano electrospray ionization source. An ACN gradient was used to elute peptides over 180 min with MS2 triggered by stepped collision energy (HCD) set to 25 and 33. The exclusion time was 30 s with a top 12 acquisition strategy. Four biological replicates were performed for each sample.

Raw files were queried against putative proteins derived from the *P. sativum* genome v1a (Kreplak *et al*., 2019) downloaded from https://urgi.versailles.inra.fr/Species/Pisum, using MaxQuant (v.2.5) (Tyanova *et al*., 2016a). Default settings were used with variable modifications oxidation (M), phospho (STY) and fixed modification carbamidomethyl (C) enabled. Peptide matching was enabled with default settings. The results generated from MaxQuant were analyzed using Perseus (v.1.6), which is a companion software to MaxQuant used for statistical analysis (Tyanova *et al*., 2016b). Phosphopeptide intensity values were loaded as main columns and decoys and contaminants were filtered. Intensity values were transformed by log_2_ and filtered based on at least three non‐zero values within one sample type, followed by between‐sample normalization by subtracting the median phosphopeptide intensity. Missing values were imputed using random numbers generated from the Gaussian distribution of the existing values, but down‐shifted by 1.8 SD (width set to 0.5 SD) to mimic low‐abundance protein LFQ values more accurately, as described by Tyanova *et al*. (2016b). Significant differences were calculated using an ANOVA test and Tukey *post hoc* testing with a false discovery rate of 0.05. Phosphorylation site motif analysis was performed with WebLogo software (Crooks *et al*., 2004).

### Transcript analysis

Following treatments, single pea embryos were pulverized in a bead mill and RNA was purified using the RNeasy Plant kit (Qiagen) according to the manufacturer's instructions. DNase digestion was performed using the turbo DNase kit (Thermo Scientific). For qPCR, cDNA was created from 250 ng of RNA using the iScript cDNA synthesis kit (BioRad) and target sequences were amplified using SSOFast Evagreen Supermix (BioRad). Average N_0_ values of two technical replicates were calculated and relative mRNA expression levels were determined by normalizing the average N_0_ against the geometric mean of the N_0_ for two reference genes, *PsED1α* and ubiquitin. Primer sequences for pea seed storage protein transcripts were taken from Garneau *et al*. (2021). For RNA sequencing, libraries were prepared with the NEBNext® Ultra™ II RNA Library Prep Kit as recommended by the manufacturer and sequenced at the Global Institute for Food Security (Saskatoon, Canada) on a NovaSeq 6000 platform (Illumina, San DIego, CA, USA). At least 19 M reads were obtained for each library. Raw sequencing data (150 bp paired‐end reads) were deposited at the NCBI Sequence Read Archive under project number PRJNA1251708. Adapters were trimmed using bbduk (parameters: ktrim = r k = 23 mink = 11 hdist = 1 tpe tbo ftm = 5) from the bbmap package (https://sourceforge.net/projects/bbmap/) and sequence quality checked using FastQC v0.12.0 (https://www.bioinformatics.babraham.ac.uk/projects/fastqc/). Trimmed reads were aligned to the *Pisum sativum* v1a genome sequence using Star v.2.7.11b (Dobin *et al*., 2012), and transcript read counts were obtained using Rsem v.1.3.3 (Li & Dewey, 2011) (parameters: ‐p 24). Transcripts per million counts were obtained and differential gene expression analysis carried out using the R package DESeq2 1.44.0 (Love *et al*., 2014). GO term enrichment analysis was conducted using the enricher function in the R package clusterProfiler v4.6.2 (Wu *et al*., 2021) (parameters: pvalueCutoff = 0.01 minGSSize = 5 maxGSSize = 500) with differentially expressed genes (log_2_fold change > 0.4 or < −0.4 and *P*adj < 0.05) and GO terms identified using the EggNOGG‐mapper tool in OmicsBox (BioBam, Valencia, Spain) as the input.

### Quantification of ABA

Procedures for the extraction, purification and quantification of ABA were previously described (Hussain *et al*., 2024). Briefly, pooled freeze‐dried embryo samples were reconstituted with methanol containing 1% acetic acid and placed at 4°C in darkness overnight. After centrifugation, d_6_‐ABA (Olchemim, Olomouc, Czech Republic) was added to the supernatants as an internal standard. Samples were dried under vacuum, reconstituted with aqueous 1% acetic acid and subjected to solid‐phase extraction using Iris‐N cartridge columns (Canadian Life Science Inc., Peterborough, ON, Canada). The ABA fraction was eluted with methanol containing 1% acetic acid. Eluates were dried in a Speedvac, reconstituted with aqueous 1% acetic acid and subjected to liquid chromatography‐electrospray ionization‐tandem mass spectrometry (Agilent 6410 series) equipped with a Zorbax C10 column (50 × 2.1 mm, 1.8 μm). MS/MS transitions, 269/159 for d6‐ABA and 263/153 for ABA, were monitored.

## Results

### Gln is the most potent amino acid stimulator of TOR in Arabidopsis leaves

**Fig. 1:**
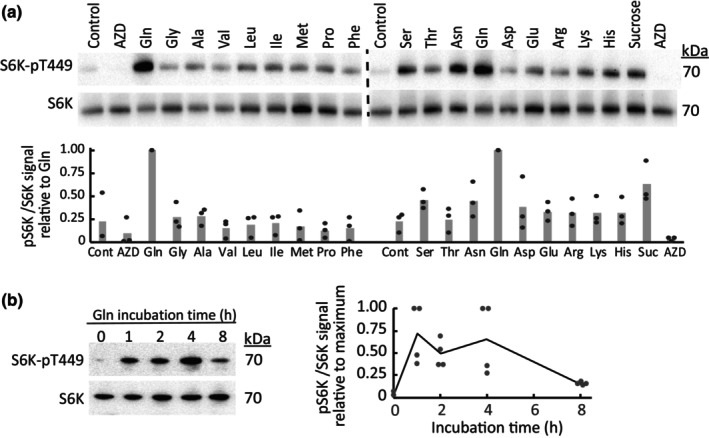
Survey of amino acid activation of target of rapamycin (TOR) activity in mature Arabidopsis leaves. (a) *S6K‐HA* leaf discs were incubated for 4 h in the dark on liquid media in the presence of individual amino acids (10 mM), sucrose (20 mM) or AZD (2 μM), followed by urea‐SDS‐PAGE and immunoblotting. Quantification of the anti‐S6K‐pT449 vs anti‐S6K signal ratio is shown relative to Gln treatments (*n* = 3). (b) Nighttime timecourse incubation of *S6K‐HA* leaf discs in media containing 10 mM Gln followed by urea‐SDS‐PAGE and immunoblotting. Quantification of anti‐pT449‐S6K to anti‐S6K signals is shown relative to the maximal signal within each experimental replicate (*n* = 4). Immunoblots from replicate experiments are shown in Supporting Information Fig. S1.

The upstream regulation of TOR may differ substantially between plant tissues with different nutritional requirements. Previously, we observed that exogenous Gln or Ile stimulated nocturnal TOR activity in mature Arabidopsis leaf tissue (O'Leary *et al*., 2020). Here, we determined the relative effectiveness of other proteinaceous amino acids in activating leaf TOR signaling using the phosphorylation status of Thr449 on ribosomal protein S6 Kinase (S6K) in an *S6K‐HA* overexpression line as a readout (Fig. 1a). In all assays, Gln was consistently the strongest nutrient activator of TOR in leaf discs (Fig. 1a). Beyond Gln, the relative ranking of TOR stimulation by other amino acids was not consistent across experiments. Trp and Tyr were not tested because they lack sufficient solubility, whereas Cys treatment was omitted because it interferes with mitochondrial respiration (Oh *et al*., 2023). Sucrose displayed less TOR activation than Gln even at a concentration of 20 mM. Treatment with the TOR inhibitor AZD5088 (AZD) completely blocked S6K‐Thr449 phosphorylation and served as a negative control. Time course experiments indicated that TOR activation by exogenous Gln was transient, being maximal between 1 and 4 h of incubation before decreasing at 8 h (Fig. 1b).

In contrast to leaf discs, TOR‐S6K‐RPS6 signaling in both the root and shoot tissues of 1‐wk‐old Arabidopsis seedlings grown on nitrate‐containing media is strongly activated by either light or sucrose but insensitive to Gln treatments (Supporting Information Fig. S2), as previously reported (Xiong *et al*., 2013). We did not observe a significant synergistic effect of Gln on sucrose activation of TOR in seedling root or shoot tissues, which has been reported in N‐starved Arabidopsis seedlings (Ingargiola *et al*., 2023). Therefore, we concluded that there are developmental and conditional differences in exogenous Gln activation of TOR in non‐starved Arabidopsis shoot tissue.

### Activation of leaf TOR‐S6K‐RPS6 signaling by Gln is dependent on darkness

**Fig. 2:**
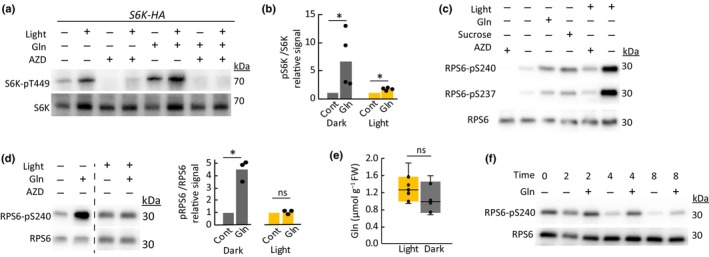
The influence of light on Arabidopsis leaf Gln‐TOR signaling. (a) S6K‐Thr^449^ phosphorylation status in *S6K‐HA* leaf discs incubated for 4 h in the light or dark in the presence or absence of 10 mM Gln or 2 μM AZD. (b) Quantification of immunoblots of Gln‐induced S6K phosphorylation. Includes data from **Figs 1(a), 2(a) and Supporting Information Fig. S3(A),** normalized to respective light or dark control treatments. Asterisks indicate significant differences (*P* < 0.05, *t*‐test; *n* = 4). (c) RPS6‐Ser^240^ and ‐Ser^237^ phosphorylation status in wild‐type leaf discs incubated for 4 h in the presence or absence of 10 mM Gln, 20 mM sucrose or 2 μM AZD. (d) Gln induction of RPS6‐Ser^240^ phosphorylation following 4 h incubations in the light and the dark. Blots were developed separately due to the much higher signal intensity in the light. Quantification of repeated measurements is shown against the respective light or dark control. Asterisks indicate significant difference (*P* < 0.05, *t*‐test; *n* = 3). (e) Gln levels in leaves harvested at 4 h into the light or dark period (*t*‐test; *n* = 6). Box plot horizontal lines indicate the median, and whiskers designate the range; internal and outlier data points are shown. (f) RPS6‐Ser^240^ phosphorylation status following timecourse incubations of leaf discs with or without 10 mM Gln at night. Immunoblots from replicate experiments are shown in Fig. S3. ns, non‐significant; TOR, target of rapamycin.

We next investigated the effect of light on nutrient‐TOR signaling in mature leaves. Exposure to light on its own enhanced S6K phosphorylation in a TOR‐dependent manner (Fig. 2a). In the light, Gln only slightly enhanced S6K phosphorylation compared to the large stimulation by Gln observed in the dark (Fig. 2a,b). The phosphorylation of ribosomal protein S6 (RPS6) at Ser237 and Ser240 is a second widely used assay of TOR activity (Dobrenel *et al*., 2016; Enganti *et al*., 2018), which we found easier to perform in mature leaves because of higher and more stable levels of RPS6 expression compared to S6K‐HA. Both Ser237 and Ser240 phosphorylation were induced by Gln and sucrose in the dark; however, light exposure resulted in comparatively greater RPS6 phosphorylation (Fig. 2c,d). As both RPS6 Ser237 and Ser240 demonstrated the same phosphorylation pattern, further analysis was restricted to Ser240 only. Gln did not induce greater phosphorylation of RPS6‐Ser240 in leaf discs exposed to light, though this residue may already be near maximal phosphorylation status in the light (Fig. 2d). Notably, Gln levels were not significantly different in daytime or nighttime leaves sampled before the start of incubations (Fig. 2e). Previous observations established that RPS6‐S240 phosphorylation displays diurnal variation, becoming less phosphorylated throughout the night (Enganti *et al*., 2018). Against this diurnal variation, the relative induction of RPS6‐S240 phosphorylation by Gln treatments during nighttime was maximal after a 4‐h incubation (Fig. 2f), which was similar to the corresponding Gln‐S6K phosphorylation dynamic (Fig. 1b). Together, these results indicate that in mature Arabidopsis leaf tissue, Gln induces a transient activation of TOR that is only readily observable in the dark when the TOR‐S6K‐RPS6 pathway is less activated.

### TOR activity influences leaf amino acid metabolism differently in light vs dark

Inhibition of TORC1 signaling strongly increases amino acid levels in plants (Moreau *et al*., 2012), and amino acid levels influence TORC1 signaling (Figs 1, 2). However, the effects of light‐ and Gln‐TOR signaling on amino acid biosynthesis and replenishment have not been examined. To attempt this, leaf discs were incubated for 6 h with uniform ^15^N‐labeled NH_4_NO_3_ alongside AZD and/or Gln treatments in either the light or dark. Subsequent GC‐MS metabolomic analysis of treatment effects focused on either ^15^N‐labeled amino acids (which were newly synthesized from exogenous N) or unlabeled amino acids, which were pre‐existing or synthesized from stored N (Figs S4, S5; Table S1).

First, light had a large effect on amino acid levels. Across all treatments, the absolute levels of labeled amino acids were overall much higher in the dark, except for Gly and Thr, which showed markedly higher labeling in the light (Table S1). Thus, assimilation of external N appeared greater at night. By contrast, the absolute levels of unlabeled amino acids (and total amino acids) were much higher in the light. As N assimilation is known to occur in the light, we hypothesize that *de novo* amino acid synthesis in daytime Arabidopsis leaves may rely on unlabeled N sources such as vacuole nitrate or NH_3_ released by the photorespiratory cycle and therefore may not be properly captured by our labeling experiment (Matt *et al*., 2001; Yoneyama & Suzuki, 2020).

**Fig. 3:**
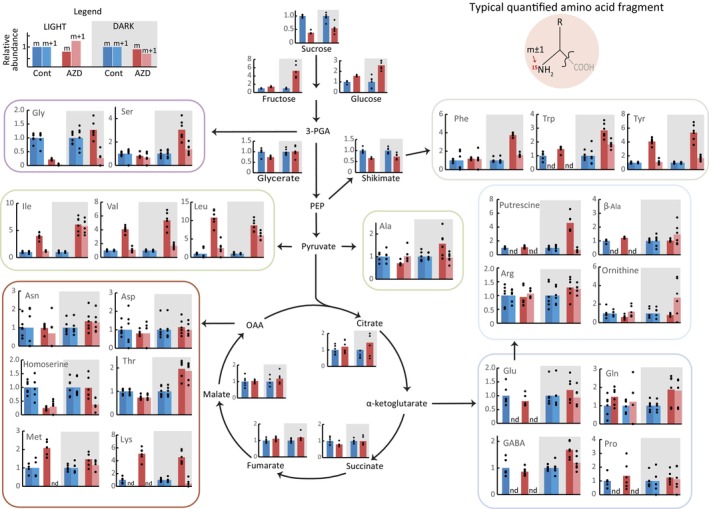
The relative abundance of ^15^N‐labeled and unlabeled metabolites in Arabidopsis leaf discs following 6 h incubations in 10 mM ^15^NH_4_^15^NO_3_ with or without 2 μm AZD in either the light or dark. All values are expressed relative to respective unlabeled (M) or labeled (M + 1) control levels in the light or dark. Labeled amino acid signals were omitted for GABA, Pro, Trp, β‐Ala and Lys in the light, as they were below a reliable detection limit. 3‐PGA, 3‐phosphoglycerate; PEP, phosphoenolpyruvate.

Second, AZD treatments strongly altered levels of both labeled and unlabeled amino acids in a light‐dependent manner. In line with previous studies, AZD treatment increased the levels of many unlabeled amino acids in both the light and the dark (Fig. 3). By contrast, nighttime AZD treatment, but not daytime treatment, increased certain labeled amino acids, namely Gln, Thr, branched‐chain amino acids and aromatic amino acids. These results indicate that the assimilation of external inorganic N was upregulated by TOR inhibition only at night; the effect of TOR on N assimilation in the day cannot be ascertained, as described above. Notably, both labeled and unlabeled Gly levels were strongly diminished by AZD in the light but not the dark, implying a strong perturbation of photorespiratory flux (directly or indirectly) by TORC1 inhibition.

Third, compared to AZD treatments, the effect of TOR activation by Gln on leaf amino acid levels was less pronounced. Because Gln supplementation could influence metabolism independently of TOR activity, we focused on Gln‐dependent effects that were reversed by the co‐addition of AZD. This pattern manifested with the Gln‐dependent increase in labeled and unlabeled Gly in the light (Fig. S4), again implicating TOR‐dependent changes to photorespiratory metabolism. In the dark, where Gln activates TOR and exogenous N assimilation is apparent, Gln treatment modestly but significantly lowered the amount of labeled Ala, Val, Lys and GABA, but only Val labeling was reversed by the co‐addition of AZD (Fig. S5). Overall, Gln and AZD treatments elicited opposing effects in specific areas of amino acid metabolism. However, the mild metabolic effects of Gln treatments did not in general contrast with the effects of AZD.

### TOR activity influences protein synthesis rate in both light and dark

**Fig. 4:**
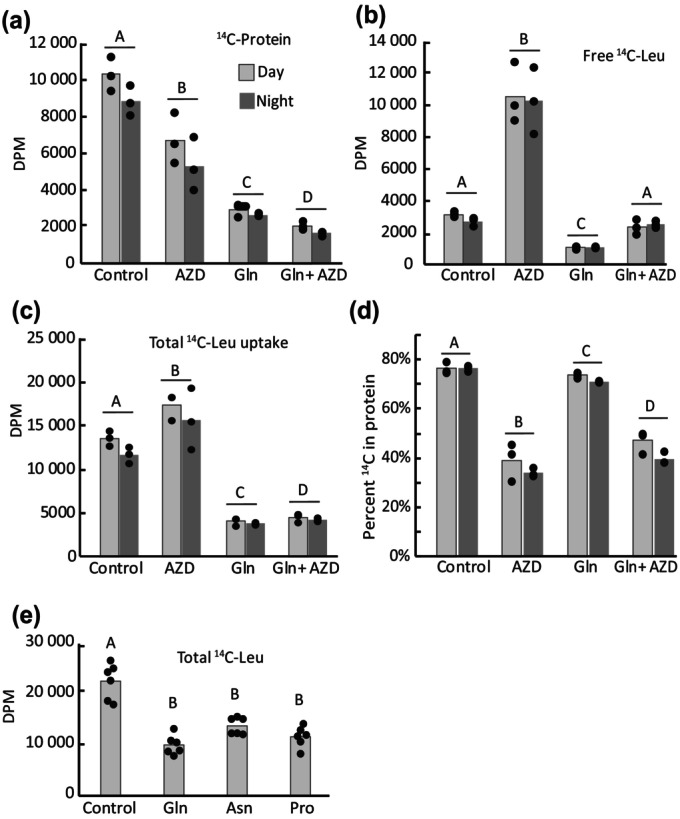
AZD inhibits total protein synthesis rates in Arabidopsis leaves during day and night. (a–d) The uptake and incorporation of Leu into protein is measured following a 6 h incubation of leaf discs with ^14^C‐Leu in the presence or absence of 10 mM Gln and 2 μM AZD (n = 3). (a) The amount of radiolabel in the protein fraction. (b) The amount of radiolabel in the free amino acid fraction. (c) The total uptake of radiolabel. (d) The proportion of imported radiolabel that becomes incorporated as protein. (e) Total uptake of ^14^C‐Leu into leaf discs following a 6 h incubation in the presence of 10 mM Gln, Asn or Pro (*n* = 6). Letters indicate significant differences between groups (ANOVA; Tukey *post hoc* analysis; *P* < 0.05). DPM, dissociations per minute.

To assess the influence of TOR activity on protein synthesis rates in mature leaves, we monitored the uptake and incorporation of ^14^C‐Leu into the protein fraction of leaf discs. AZD treatments in either light or dark decreased Leu incorporation into protein by 35% compared to control treatments (Fig. 4a), while simultaneously increasing total uptake of external Leu and the accumulation of free Leu (Fig. 4b,c). Therefore, the fraction of imported Leu that was incorporated into protein was strongly decreased by AZD (Fig. 4d). By contrast, the main effect of Gln treatment was to block the total uptake of external Leu. This effect was only slightly mitigated by the co‐addition of AZD and was therefore independent of TOR signaling. Other amino acid treatments, besides Gln, also limited ^14^C‐Leu uptake (Fig. 4e). Altogether, TOR inhibition limited total protein synthesis rate in mature leaves similarly in the light and dark, but the effect of TOR activation by Gln on protein synthesis could not be assessed because Gln supplementation impaired Leu uptake.

### Gln is the sole exogenous nutrient activator of TOR in developing pea cotyledons

**Fig. 5:**
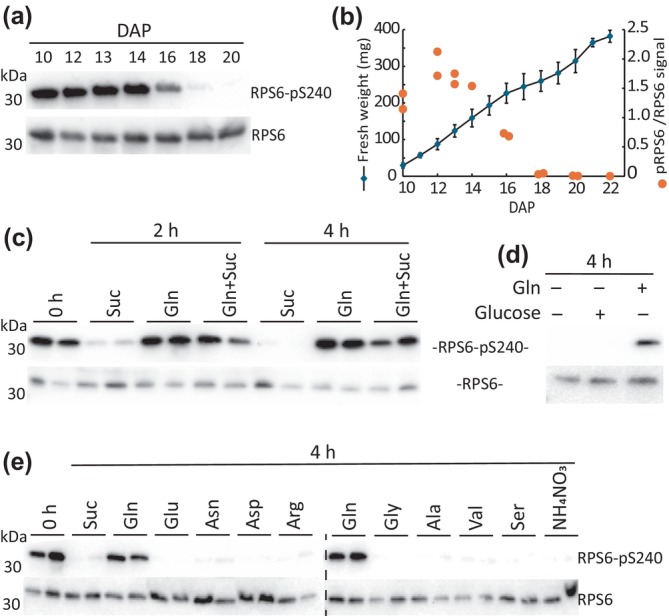
Gln activates target of rapamycin (TOR) in developing pea cotyledons. (a) The *in vivo* RPS6‐Ser^240^ phosphorylation status in pea embryos throughout development. Replicate immunoblots are shown in Supporting Information Fig. S5. (b) The relative RPS6‐Ser^240^ phosphorylation signal is plotted, and the fresh weight of the embryos is plotted against days after pollination (DAP). Error bars represent the standard error of the mean. (c–e) RPS6‐Ser^240^ phosphorylation was assessed in 90–130 mg pea embryos incubated for 2 or 4 h with or without Gln (62.5 mM) and sucrose (142 mM) (c), Gln (62.5 mM) and glucose (100 mM) (d) or individual amino acids (62.5 mM) and NH_4_NO_3_ (20 mM) (e). Two biological replicates are shown. Replicate immunoblots are shown in Fig. S6.

To determine whether amino acids influenced TOR during seed development, we switched from Arabidopsis, where the scale of materials is technically limiting, to pea, where embryos are larger. To our knowledge, nutritional regulation of TORC1 in developing seeds has not yet been established in any plant species. By analyzing the *in vivo* RPS6‐S240 phosphorylation status of pea embryos sampled directly from the plant at midday, we observed a developmental profile wherein TORC1 activity was relatively high until embryos reached 150 mg FW (approximately half their final weight), after which TORC1 activity decreased rapidly to undetectable levels (Fig. 5a,b). This pattern coincides with the known occurrence of mitotic activity in the developing embryo (Weber *et al*., 2005). Developing pea embryos can be cultured *in vitro* when supplied with sucrose and amino acids, which are the natural macronutrients supplied from maternal tissues (Thompson *et al*., 1977). Using this system, we tested whether the specific nutrients maintain TORC1 activity in cultured pea embryos at the 90–130 mg stage corresponding to 12–13 d after pollination (DAP) when background TOR activity is high. In a background media consisting of modified, nitrogen‐free MS salts, the phosphorylation of RPS6‐S240 was dependent solely on the addition of Gln and not sucrose (Fig. 5c). Similarly, additions of glucose, other amino acids or NH_4_NO_3_ did not maintain RPS6‐Ser240 phosphorylation (Fig. 5d,e). The omission of the MS salts from the media did not reduce RPS6‐Ser240 phosphorylation when Gln was present (Fig. S6D). Sufficient osmotic potential is important for culturing pea embryos in media; however, the presence or absence of 5% (w/v) sucrose or mannitol in the media did not appreciably affect RPS6 phosphorylation in the short term (4 h) when Gln was present (Fig. S6E). During 4 h incubations, embryo sucrose content was maintained by 5% sucrose in the media but decreased markedly in its absence, demonstrating functional sucrose transport during embryo culture (Fig. S6F). Therefore, among exogenous nutrients, only Gln could stimulate RPS6‐Ser240 phosphorylation in developing pea embryos.

**Fig. 6:**
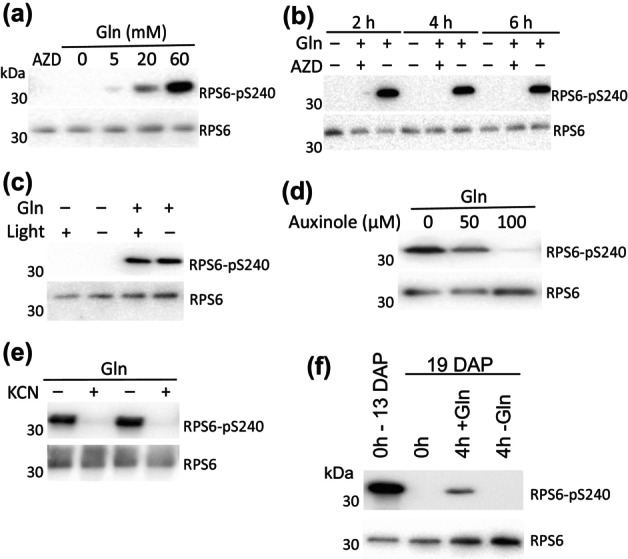
Multiple non‐nutritional factors control target of rapamycin (TOR) activity in developing pea embryos. RPS6‐Ser^240^ phosphorylation status was determined following 4 h incubations of 90–130 mg pea embryos in the light in culture media containing MS salts, 5% sucrose (w/v) and 62.5 mM Gln, with modifications as indicated. (a) Incubations with various concentrations of Gln or 2 μM AZD. (b) Timecourse incubations with or without Gln and 2 μM AZD. (c) Incubations with and without Gln in the light and dark. (d) Incubations with the auxin signaling inhibitor auxinole. (e) Incubations with the respiratory inhibitor KCN^−^ (2 mM). (f) Incubations of older 300 mg (19 DAP) embryos with and without Gln. Replicate immunoblots are shown in Supporting Information Fig. S6. DAP, days after pollination.

The effect of external Gln on RPS6‐S240 phosphorylation increased sharply with concentrations up to 60 mM (Fig. 6a). We determined sucrose and Gln concentrations in the liquid endosperm surrounding young embryos to be 124 ± 4 mM (*n* = 6) and 58 ± 4 mM (*n* = 4), respectively. Within embryos, Gln levels decreased approximately linearly throughout development, starting from 10 mg g^−1^ FW at 10 DAP, which would convert to 68 mM Gln if the FW consisted entirely of water (Fig. S7A). Sucrose levels also decreased during embryo development, though less dramatically than Gln (Fig. S7B). Together, these results indicate that variations in Gln‐TOR signaling can occur at physiologically relevant concentrations of Gln in pea embryos. In contrast to our observations in Arabidopsis leaf tissue, Gln‐dependent RPS6‐S240 phosphorylation in pea embryos was both non‐transient (Fig. 6b) and independent of light (Fig. 6c). However, Gln‐induced TORC1 activity in pea embryos was completely blocked by the auxin signaling inhibitor auxinole (Fig. 6d) and by the respiratory inhibitor cyanide (Fig. 6e). Lastly, exogenous Gln was much less effective at stimulating TOR activity in older embryos compared to younger embryos, suggesting that *in vivo* downregulation of TOR activity during development might depend on additional factors besides Gln levels (Fig. 6f, compare with 5c). Therefore, despite its narrowed regulation by nutritional factors, the TOR‐S6K‐RPS6 signaling axis in developing pea embryos still integrates multiple other regulatory inputs.

Given that TOR signaling involves phosphorylation cascades that extend beyond S6K and RSP6, we investigated other targets of Gln‐TOR signaling in developing pea embryos by a phosphoproteomic analysis. Pea embryos weighing *c*. 130 mg were cultured in MS media with 5% sucrose and either Gln (62.5 mM), AZD (2.5 μM) or Gln and AZD for 3 h (Table S2). We identified 188 differentially abundant phosphorylation sites between the TOR‐activated (Gln) and inactivated states (AZD or Gln + AZD) on direct or indirect target proteins (Fig. S8; Table S3). GO ontology analysis of the putative target proteins indicated categorical enrichment of processes related to translation and autophagy (Table S4). TOR‐dependent phosphosites were also enriched for a proline‐directed phosphorylation motif (Fig. S8D). Peptides representing 38 phosphosites were significantly different in abundance between the Gln and both AZD‐containing treatments and were thus higher confidence identifications (Table 1). Among these targets were both established TORC1 targets such as RPS6, LARP1, ATG13 and EIN2 (Van Leene *et al*., 2019; Scarpin *et al*., 2020; Fu *et al*., 2021) as well as novel putative downstream targets including a demonstrated eIF4E binding protein EXA1, and the vacuolar amino acid transporter YPQ1. Several AZD‐dependent phosphosites also contained SnRK1 phosphorylation motifs, including a known SnRK1‐dependent phosphorylation site on fructose‐6‐phosphate‐2‐kinase/fructose‐2,6‐bisphosphatase (Table S3). Seed‐specific targets were also identified such as the AMP deaminase, EMBRYONIC FACTOR‐1 and the condensin complex subunit, EMBRYO‐DEFECTIVE‐1. While new putative TOR substrates require further investigation, we concluded that TORC1 signaling in developing pea seeds regulated many typical TOR‐dependent functions such as translation, autophagy and hormonal signaling.

**Table 1:** List of significant phosphorylation site differences (*P* < 0.05) detected in comparisons between both Gln and AZD and Gln and Gln + AZD treatments of pea embryos.

Average fold change^1^	Pea gene	Nearest Arabidopsis homolog	Abbreviated protein description^2^	Phosphopeptide	Prev. I.D.^3^
7.02	Psat7g115800	RPS6B	Ribosomal protein S6e	**(pS)** ^**237**^RL**(pS)** ^**240**^TATKPTVAA	[1,2]
5.79	Psat1g204040	RPS6B	Ribosomal protein S6e	**(pS)** ^**237**^RLSTAKPVAA	[1,2]
5.17	Psat2g021800	LARP1a	LA‐RELATED PROTEIN 1A	LSSSPHGHGGLSGSSPPVG**(pS)** ^**749**^LPK	[1,2]
3.30	Psat0s1379g0200	AT3G24080	KRR1 family protein	EKTEI**(pT)** ^**453**^ **(pS)** ^**454**^DDDVVE**(pS)** ^**461**^ENEKEEIPDEGSSR	
3.12	Psat3g031640	ATS40‐7	Senescence regulator	TTF**(pS)** ^**191**^VLEGVGR	[1]
2.86	Psat6g045440	AT1G06620	Aminocyclopropane‐1‐carboxylate oxidase homolog	**(pS)** ^**2**^ **(pS)** ^**3**^PIATTLSSPPYDR	
2.84	Psat5g055120	ML1	MEI2‐LIKE PROTEIN 1	GNVNDLTSSVGQG**(pS)** ^**673**^PK	[1]
2.72	Psat0s3731g0120	EXA1	ESSENTIAL FOR POTEXVIRUS ACCUMULATION 1; eIF4E BP	LLSS(**pS**)^**1581**^PVSSQSSQK	
2.70	Psat5g055120	ML1	MEI2‐Like Protein 1	QHSYLGE**(pS)** ^**623**^PDAPGFR	[1]
2.51	Psat2g162440	AT4G36850	Vacuolar amino acid transporter YPQ1	SIPSRPEYYYG**(pS)** ^**170**^AR	
2.32	Psat5g041240	FAC1	AMP deaminase; EMBRYONIC FACTOR 1	**(pS)** ^**96**^VDENSMNLLR	[2]
2.26	Psat2g162440	AT4G36850	Vacuolar amino acid transporter YPQ1	SLAGNM**(pT)** ^**179**^PPSR	
2.14	Psat2g021800	LARP1a	LA‐RELATED PROTEIN 1A	SQSHTSGASNINPGENAVG(**pS**)^**657**^VEESGGNNSR	[1,2]
2.13	Psat2g061760	ATG13A	Autophagy‐related protein 13	IITDYVG**(pS)** ^**234**^PNTDPLR	[1]
1.70	Psat7g241920	ATG1C	Serine/threonine‐protein kinase ATG1‐like	DLTNPLG(**pS**)^**396**^PEQIFANPYPK	[1]
1.69	Psat7g203760	EMB2795	EMBRYO‐DEFECTIVE 2795	IQ**(pS)** ^**20**^PTSPFLLGSNDDQLER	
1.32	Psat3g122440	RAPTOR1	Regulatory‐associated protein of TOR 1	TIEATISP(**pS**)^**912**^LAR	[2]
1.14	Psat6g193320	EIF5B1	Eukaryotic translation initiation factor 5B	SEL**(pT)** ^**132**^GDGDGDEDEPVVSFTGK	[2]
0.93	Psat6g194880	ATIM	Timeless family protein	DE**(pS)** ^**1239**^DDEMLGSILK	
0.87	Psat3g192280	TOP1ALPHA	DNA topoisomerase I	FDDD**(pS)** ^**19**^DDDQPLSFK	
0.45	Psat3g022000	P23‐1	Co‐chaperone protein p23	FGGMGGADDIDE**(pS)** ^**196**^DDEGQEVSKPGEEDAGK	[2]
−0.72	Psat4g205240	KIN7.3	KINESIN 7.3; microtubule motor	**(pS)** ^**638**^VDHIDLLR	
−0.76	Psat3g041280	EIN2	ETHYLENE INSENSITIVE 2	KYH**(pS)** ^**925**^LPDISGYSIPHR	[3]
−0.91	Psat6g234240	CIP8	RING‐H2 protein; COP1‐INTERACTING‐PROTEIN 8	LIAESSREDDA**(pS)** ^**90**^PPPPPSR	
−1.11	Psat7g147000	AT3G45090	Nucleotide triphosphate hydrolase superfamily	SL**(pS)** ^**744**^IPAIR	
−1.12	Psat7g210320	SRRM1L	PWI domain‐containing splicing factor	RRI**(pS)** ^**409**^PSPMHLR	[2]
−1.29	Psat7g078600	AT1G30320	Remorin family protein	VQTGII**(pS)** ^**15**^PSK	
−1.77	Psat0s1687g0040	HMGA	HMGA (high mobility group A) protein	AKDPLA**(pS)** ^**101**^PPSGAVSTPRPR	
−2.10	Psat3g189480	CPL3	C‐terminal domain phosphatase‐like 3	DHDLDSLP**(pS)** ^**44**^PTR	
−2.32	Psat4g102840	AT4G35785	Serine/arginine‐rich splicing factor SR45a‐like	**(pS)** ^**271**^IPH**(pS)** ^**275**^PYSPDRR	
−4.81	Psat0ss3186g0200	AT4G32420	Cyclophilin type peptidyl‐prolyl *cis*‐trans isomerase	VR**(pS)** ^**601**^l**(pS)** ^**603**^R**(pS)** ^**605**^PVR	

^1^ The average log_2_ fold change in peptide abundance between Gln vs AZD and Gln vs Gln + AZD treatments.

^2^ Extended description is found in Table S3.

^3^ TOR‐dependent phosphoproteins that were previously identified by [1] Van Leene *et al*. (2019); [2] Scarpin *et al*. (2020) or [3] Fu *et al*. (2021).

### TOR and ABA signaling interact to control pea seed storage protein synthesis

**Fig. 7:**
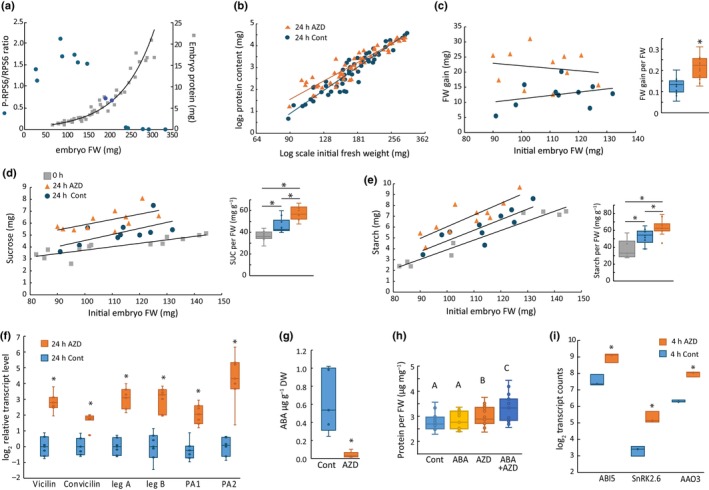
Effect of target of rapamycin (TOR) inhibition on protein synthesis and abscisic acid (ABA) signaling in developing pea seeds. (a) Embryo protein content and relative TOR activity status (from Fig. 5b) are plotted against embryo fresh weight for freshly harvested embryos. (b) Protein content after 24 h incubation of pea embryos with 10% sucrose and Gln (62.5 mM) in the presence or absence of AZD (2.5 μM) plotted against initial fresh weight. There is a significant interaction between treatment and fresh weight (ANCOVA, *P* < 0.01). (c–g) Pea embryos in early seed fill (90–130 mg) were immediately frozen (0 h) or incubated for 24 h with Gln (62.5 mM) and 10% sucrose (w/v) in the presence or absence of AZD (2.5 μM). Total FW gain (c), total sucrose (d) and total starch (e) were plotted against initial FW and compared on a per initial FW basis (*n* = 13). (f) Relative *transcript* levels of pea storage proteins were measured via qPCR (*n* = 6). (g) ABA contents were measured on a dry weight basis (*n* = 5). Asterisks indicate significant differences between treatment pairs (*t*‐test; *P* < 0.05). (h) Protein amounts were determined in 90–130 mg pea embryos following 24 h incubations with Gln (62.5 mM) in the presence or absence of 2.5 μM AZD and 5 μM ABA. Letters indicate significant differences between groups (ANOVA; Tukey *post hoc* analysis; *P* < 0.05; *n* = 12). (i) Log_2_ DESeq2 normalized counts of select ABA‐related transcripts following 4‐h incubation with Gln and in the presence or absence of 2.5 μM AZD. Asterisks indicate significant differences between treatment pairs (adjusted *P* < 0.05; *n* = 3). Pea gene references, ABI5: Psat3g033680; SnRK2.6: Psat7g123680; AAO3: Psat0s2541g0120. Box plot horizontal lines indicate the median and whiskers designate the range; internal and outlier data points are shown.

As cultured pea embryos grow and accumulate protein for at least 48 h (Fig. S9), we could also use this system to test whether seed protein synthesis was dependent on TORC1 activity. Embryo protein content depends on seed size in a non‐linear fashion, with larger, more mature seeds containing disproportionately more protein because of the developmental upregulation of seed storage protein synthesis (Fig. 7a). Here, seed storage protein accumulation and starch accumulation began at 13 DAP when pea embryos were *c*. 100–130 mg in size (Fig. S7C,D). Therefore, *in vivo* TOR activity decreased at the onset of storage protein deposition during pea embryo filling (Fig. 7a). Next, we incubated embryos at various stages of seed filling for 24 h in TOR‐activating media containing 10% sucrose and Gln in the presence or absence of AZD. Embryo fresh weights were measured before and after incubation. Unexpectedly, TOR inhibition by AZD caused an increase in protein accumulation, but only with embryos smaller than *c*. 160 mg FW (equivalent to 14 DAP) and not larger, more mature embryos (Fig. 7b). AZD treatment of 90–130 mg cultured embryos also increased embryo weight gain (fresh and dry weight), as well as sucrose and starch contents (Fig. 7c–e). Furthermore, there was a substantial increase in the transcript abundance of seed storage protein‐encoding genes in these AZD‐treated embryos compared to control embryos of matching initial FW (Fig. 7f). Therefore, in contrast to its regulatory effects in other tissues, the maintenance of TOR activity in developing peas at the onset of seed fill inhibits or delays biosynthesis in general and protein synthesis in particular.

To potentially explain this unexpected result, we note previous observations that curtailment of either SnRK1 or abscisic acid (ABA) signaling in developing pea embryos caused a developmental delay and reduced seed storage protein synthesis at seed fill (Radchuk *et al*., 2006, 2010). Furthermore, transcription of seed storage protein‐encoding genes is strongly dependent on ABA signaling (Finkelstein *et al*., 2002). Since both ABA and SnRK1 signaling are known to regulate TORC1 activity in a reciprocal fashion, we investigated whether TORC1 inhibition served to activate ABA signaling in developing pea embryos, thus activating seed storage protein synthesis. Treatment of 90–130 mg embryos with AZD caused a marked decrease in tissue ABA levels after 24 h, but not after 4 h, indicating a strong interaction between TOR activity and ABA signaling (Figs 7g, S10). Furthermore, exogenous ABA treatment had no effect on protein accumulation in these developing embryos when TORC1 was active, but when TORC1 was inhibited, exogenous ABA stimulated protein synthesis (Fig. 7h).

To clarify the interaction between TOR and ABA signaling, RNA sequencing was performed on samples of 115–130 mg embryos incubated 4 h with Gln and 10% sucrose in the presence or absence of AZD. A total of 1356 upregulated and 1652 downregulated genes were identified in response to AZD treatment (Table S5). Upregulated transcripts upon TOR inhibition were enriched for seed storage protein (Table S6) and ABA‐responsive genes, and included members of the core ABA signaling and biosynthesis pathway: *ABI5, AAO3 and SnRK2.6* (Figs 7i, S10). Established downstream targets of ABI5 in legume seeds were also upregulated, including a subset of late embryogenesis abundant genes and genes related to raffinose family oligosaccharide biosynthesis (Zinsmeister *et al*., 2016) (Table S5). Another group of AZD‐upregulated transcripts encoded UDP‐glucosyl transferases that showed homology to the ABA glucosyltransferase identified in adzuki and common bean and could potentially be involved in the conjugation and sequestration of free ABA (Palaniyandi *et al*., 2015). Notably, genes related to ribosomal and translational processes were strongly enriched among AZD‐downregulated transcripts, despite an overall increase in protein synthesis (Fig. S10). These results indicate that TORC1 inhibition activated legume seed ABA signaling and seed storage protein synthesis while lowering free ABA levels, possibly by increased ABA conjugation.

## Discussion

### Tissue‐specific patterns of TOR nutrient regulation

A growing number of nutrient, hormonal and energy status signals are known to operate upstream of TORC1 activation in plant tissues, supporting TORC1's description as a master regulator of plant metabolism, growth and development (Liu *et al*., 2021; Meng *et al*., 2022; Rabeh *et al*., 2024). However, the same contingent of upstream regulatory signals is unlikely to operate across all plant tissues, which display major differences in metabolism and development. This study demonstrates that nutrient activators of TORC1 differ across plant tissues. The carbohydrate‐based signals, glucose and sucrose, dominate TORC1 activation in Arabidopsis seedling tissues, while conversely, Gln appears to be the dominant nutrient signal activating TORC1 in darkened Arabidopsis leaves and in developing legume seeds. The situation in other seeds such as cereals and oilseeds still needs investigation.

A physiological rationale for the pattern of nutritional TORC1 activation among plant tissues is difficult to determine at present because the nature of how glucose signaling activates TOR is unclear. In animal cell culture, glucose withdrawal increases cellular AMP to ATP levels, which downregulates TORC1 via activation of AMP‐activated protein kinase (AMPK; the orthologue of SnRK1 in plants) (Gwinn *et al*., 2008; Panwar *et al*., 2023). This reciprocal regulatory network between SnRK1 and TORC1 also exists in plants to coordinate cellular energy signaling (Nukarinen *et al*., 2016; Belda‐Palazon *et al*., 2020; Jamsheer *et al*., 2022). However, in AMPK‐deficient animal cells, glucose levels still signal to TOR via the glycolytic intermediate dihydroxyacetone phosphate (DHAP) (Orozco *et al*., 2020). Likewise, in *Chlamydomonas reinhardtii*, light and carbon fixation stimulate TOR activity via the cytosolic accumulation of DHAP (Mallen‐Ponce *et al*., 2025). Returning to plants, in carbon‐starved Arabidopsis root meristems, glycolytic and respiratory inhibitors completely inhibited TOR reactivation by glucose (Xiong *et al*., 2013). This result was interpreted to mean that glucose activation of TORC1 occurred via a respiratory energy signal (likely related to ATP), but it cannot be excluded that both a carbohydrate‐related signal and an ATP‐related signal are simultaneously needed for TOR activation in some plant tissues. For comparison, in developing pea embryos, respiratory inhibition also blocked TORC1 activation by Gln (Fig. 6). Gln, however, was unlikely to be used for respiratory ATP generation, especially since sucrose was present in the media. Therefore, it is likely that Gln and ATP represent obligate yet mechanistically separate signals that converge at or above the TORC1 complex in certain plant tissues. In seedlings, changes in carbohydrate supply may be more closely linked to ATP levels than in mature tissues or seeds due to seedlings' lesser reserves of nutrients, potentially conflating carbohydrate and energy signals.

Results from this study demonstrate that amino acids are major nutrient regulators of TOR in certain plant tissues, consistent with typical nutrient regulation of TORC1 throughout eukaryotes. Regulation of plant TORC1 by amino acids is intuitive because, as in other eukaryotes, TORC1 activity strongly regulates protein and amino acid metabolism and therefore will benefit from perceiving amino acid status. The rationale behind the predominance of Gln as an amino acid activator of TORC1 in plant tissues examined so far can be considered from a metabolic standpoint. Gln and Glu serve as the central hub into which inorganic N is assimilated in plants via the combination of glutamine synthetase and glutamine‐2‐oxoglutarate transaminase (GS‐GOGAT). Following N assimilation, the amine and amide groups of Glu and Gln are distributed to create all other amino acids and N‐containing compounds in the cell (Lee *et al*., 2023). Whereas Glu concentrations in plant cells are remarkably stable across changing conditions, Gln levels can fluctuate greatly across environments, making Gln a comparatively better signaling molecule (Fritz *et al*., 2006; Forde & Lea, 2007; Mallen‐Ponce *et al*., 2025). In mammalian cells, besides Gln, TOR is particularly responsive to the amino acids Leu and Arg, which are essential and conditionally essential, respectively (Lutt & Brunkard, 2022). As plants can biosynthesize all amino acids, there is no need to detect essential amino acids *per se*. Therefore, Gln is seemingly among the best situated molecules in the plant metabolic network to convey information about the cellular organic nitrogen status (Lee *et al*., 2023). Inorganic nitrogen, namely nitrate, is also perceived by plants via separate mechanisms (Delgado *et al*., 2024).

Developing legume embryos import most of their N in the form of amino acids, and unlike roots or leaves, nitrate assimilation within developing cotyledons appears to be minimal (Chopin *et al*., 2007; Tegeder & Masclaux‐Daubresse, 2018). Gln is the most abundant amino acid in pea pod phloem delivered to the seed coat and the most abundant amino acid secreted by pea seed coats for uptake into the embryo (Christine & Boutin, 1991; Lanfermeijer *et al*., 1992; Zhang *et al*., 2015). Furthermore, the delivery of amino acids may be limiting for pea seed development, as overexpression of the major amino acid transporter (AAP1) in either source leaves or in legume seed tissues increased seed protein content and yield per plant (Rolletschek *et al*., 2005a,b; Zhang *et al*., 2015). In our current study, the Gln concentration required to fully activate TORC1 in cultured pea embryos was remarkably high at *c*. 60 mM (Fig. 6). However, this figure closely matches the Gln concentration in the liquid endosperm surrounding young pea embryos, as measured both in this study (58 mM) and previously at *c*. 60 mM (Christine & Boutin, 1991). Together, these results imply that Gln is a physiologically relevant TORC1 signal representing maternal nutrient delivery. Sucrose delivered to developing embryos is also an important nutrient signal that, while not affecting TOR activity in our experiments, can be detected by other mechanisms such as hexokinase or Tre‐6‐phosphate‐based signaling (Weber *et al*., 2005; Meitzel *et al*., 2021; Pegler *et al*., 2023). It may also be the case that carbohydrate levels are so high in the developing pea embryo (with liquid endosperm containing 124 mM sucrose) that a disruption in external sucrose supply has little short‐term effect on any cytosolic carbohydrate‐dependent signals.

While only Gln maintained TORC1 activation in developing seeds, multiple exogenous amino acids activated TOR in mature leaves, as they do in N‐starved seedling shoots (Liu *et al*., 2021) (Fig. 1). Furthermore, in leaves but not embryos, Gln‐TOR signaling (Fig. 2) was both transient and light dependent. The photorespiratory N cycle is by far the largest N flux in photosynthesizing leaf cells, wherein ammonia released by the glycine decarboxylase complex is reassimilated by glutamine synthetases at rates as much as 10‐fold higher than *de novo* ammonia assimilation (Oliveira *et al*., 2002). We speculate that the presence of this amino acid flux may have necessitated adaptations with regard to how amino acids interact with the TOR network and may in part explain the insensitivity of TORC1 activity toward Gln in the light. By contrast, developing green embryos, while photosynthetically competent, display low photorespiratory fluxes due to low gas exchange, causing a low O_2_ and high CO_2_ environment (Rolletschek *et al*., 2005a,b; Schwender *et al*., 2006). Further research will be required to understand how TORC1 regulation has been integrated into distinct modes of plant nitrogen metabolism, including photorespiratory metabolism.

The reason for differences in Gln‐TOR signaling properties across plant tissues is unclear, and we cannot exclude the possibility of multiple distinct mechanisms of Gln detection upstream of TORC1. Although Gln activation of TORC1 is highly conserved across eukaryotes, the mechanism of Gln sensing has not been decisively determined. Rather, multiple non‐exclusive mechanisms have been proposed for animal and yeast cells (Duran *et al*., 2012; Bernfeld *et al*., 2018; Meng *et al*., 2020; Jin *et al*., 2024). In yeast, the protein Pib2 is proposed to be a direct glutamine sensor that binds and activates TORC1 (Tanigawa & Maeda, 2017). Recently, FYVE1/FREE1 was identified to be an orthologue of Pib2 in plants and was demonstrated to activate TORC1 in a Gln‐dependent fashion (Tanigawa *et al*., 2024). In addition, the activation of TOR by exogenous inorganic nitrogen and amino acid treatments in nitrogen‐starved Arabidopsis seedlings was shown to involve the GTPase ROP2, which also mediates TOR activation by auxin (Schepetilnikov *et al*., 2017; Liu *et al*., 2021). Gln is also sensed in most plants (though not Brassicaceae) by the plastid‐localized PII protein, but PII's regulatory effects appear limited to fatty acid and Arg biosynthesis, and no connection to TORC1 has yet been reported (Chellamuthu *et al*., 2014; Lee *et al*., 2023). Given that embryonic Gln‐TOR signaling operated independently of light and carbohydrate supply, developing pea seeds (and potentially other seeds) offer a simplified system for future biochemical studies on the mechanism of Gln detection upstream of TORC1.

### TOR and ABA coordinate storage protein synthesis in developing pea embryos

Our results revealed a unique regulatory role for TORC1 in developing pea seeds. The stimulation of protein synthesis rate is one of the best‐established effects of TORC1 activation across eukaryotes. For example, AZD treatment decreased protein synthesis rates in Arabidopsis leaf discs (Fig. 4). However, the opposite result was observed in developing pea seeds, wherein AZD treatment increased protein synthesis in embryos at early seed fill (Fig. 7). Phosphoproteomic data indicated that TOR inhibition in developing pea seeds affected many established molecular targets including translational regulators (Table 1). Transcriptomic analysis confirmed that many transcripts related to ribosome biogenesis and translation were downregulated upon TOR inhibition (Fig. S10). There was no evidence that the nature of downstream TOR signaling toward protein synthesis was drastically altered in developing pea embryos. Rather, the control of storage protein synthesis in developing legume seeds is atypical because it is upregulated by the stress response‐promoting hormone ABA and occurs following embryonic cellularization during seed desiccation (Weber *et al*., 2005; Radchuk *et al*., 2010). Thus, legume seed storage proteins are made in response to stress signals during increasing desiccation stress, whereas typical eukaryotic protein synthesis is inhibited by environmental stress (Spriggs *et al*., 2010).

We observed that TORC1 activity displays a clear developmental profile in developing pea embryos: high TOR activity in early development decreases to zero at the onset of the prolonged seed filling phase (Fig. 7a). The maturation of legume embryos is marked by a transition of cotyledon cells from a mitotically active state to a differentiated state (Weber *et al*., 2005). Storage protein synthesis occurs in differentiated cells and not in mitotically active cells. This transition does not occur uniformly in all cotyledon cells; rather, a spatial developmental gradient exists wherein adaxial cotyledon cells close to the embryonic axis are more developmentally advanced (Weber *et al*., 2005). We observed that the stimulatory effect of TORC1 inhibition on protein accumulation was age dependent, occurring most in relatively younger embryos, which contain more undifferentiated cells, and disappearing in older embryos, which contain mostly differentiated cells (Fig. 7b). Therefore, our data support the hypothesis that TORC1 inhibition accelerated the transition of undifferentiated cotyledon cells into the differentiated state, thereby hastening the onset of storage protein synthesis in these cells and causing greater protein synthesis overall, at least in the short term. As demand for Gln would increase during seed storage protein synthesis, we must conclude that TORC1 no longer functions to integrate amino acid supply and demand during pea seed fill. We observe that TOR becomes developmentally inactivated during seed fill, and it is unclear how seed storage protein synthesis is coordinated with amino acid levels. The purpose of Gln as a signal upstream of TOR in young embryos before seed fill requires further study. Does it solely serve to integrate cellular supply and demand? Genetic loss of TORC1 causes Arabidopsis embryo abortion at a very early stage (Menand *et al*., 2002; Ren *et al*., 2011). The embryonic tissues examined in this study were comprised of a mixture of mitotic and differentiated cells, and future experiments should investigate the functions of Gln‐TOR signaling in younger mitotically active embryos, which may abort in response to TOR inhibition rather than differentiate.

In pea seeds, TORC1 activity interacted strongly with ABA signaling, as has been observed in other tissues (Fig. 7g–i; Table S5). In contrast to TORC1 activity, ABA levels peak at the onset of the filling phase in pea, and ABA signaling promotes the expression of seed storage proteins (Radchuk *et al*., 2010). ABA and TORC1 signaling pathways are known to reciprocally regulate each other, with SnRK1 activation being a major component of the ABA signaling (Wang *et al*., 2018; Belda‐Palazon *et al*., 2020, 2022). Active TORC1 phosphorylates and inactivates PYL family ABA receptors (Wang *et al*., 2018). On the other hand, ABA signaling promotes SnRK2‐ and SnRK1‐dependent inhibition of TORC1 via phosphorylation of RAPTOR (Wang *et al*., 2018; Belda‐Palazon *et al*., 2020). Disruption of either ABA or SnrK1 signaling in developing pea embryos delays maturation and lowers protein accumulation (Radchuk *et al*., 2006, 2010), thus opposing the effect of TORC1 inhibition observed here. We noted that TORC1 inhibition strongly decreased ABA levels in cultured pea embryos, while simultaneously elevating the expression of storage protein and ABA signaling genes (Fig. 7). A similar pattern was observed in Arabidopsis vegetative tissues where TORC1 disruption caused hypersensitivity to ABA alongside a decrease in ABA levels (Deprost *et al*., 2007; Kravchenko *et al*., 2015; Salem *et al*., 2018). These results suggest that in seeds, as elsewhere, TORC1 inhibition promotes ABA signaling, but this leads to an eventual recalibration of ABA levels (Salem *et al*., 2018). Consistent with this, exogenous ABA stimulated protein synthesis only in AZD‐treated embryos, indicating that sensitivity to ABA is important in controlling legume seed storage protein synthesis (Fig. 7h).

**Fig. 8:**
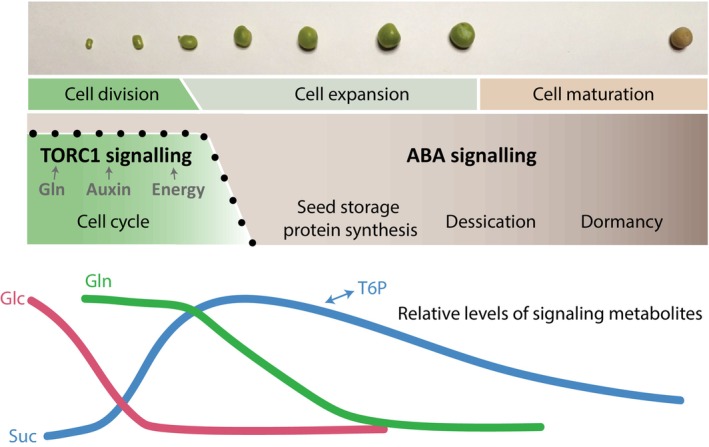
Hypothetical model showing the reciprocal regulation of target of rapamycin (TOR) and abscisic acid (ABA) signaling during the transition from the cell division to the cell expansion phase of legume seed development. The activators of TOR observed in this paper are shown, but further molecular and hormonal influences on the developmental transition are omitted for simplicity. The bottom panel extends a basic model by Weber *et al*. (2005) to show the approximate timing of relative changes in three established signaling metabolites: Glc, glucose; Suc, sucrose; and Gln, glutamine. Sucrose levels are known to be correlated with the signaling metabolite T6P.

Altogether, we hypothesize that in legume embryos the interplay between TOR, ABA and SnRK1 signaling exerts control on the timing of the transition from mitotically active cells, which TORC1 supports, to differentiated storage compound accumulating cells, which ABA signaling supports (Radchuk *et al*., 2006) (Fig. 8). A parallel nutrient signaling axis based upon the sucrose‐trehalose 6‐phosphate (T6P) nexus and its effect on auxin biosynthesis has also been discovered to influence this developmental transition, as well as starch synthesis in pea (McAdam *et al*., 2017; Meitzel *et al*., 2021). The known regulation of SnRK1 activity by T6P levels is a potential bridge between carbohydrate levels and TOR activity in legume seeds that requires further investigation (Van Leene *et al*., 2022; Morales‐Herrera *et al*., 2024). Understanding the control of this transition is important because cotyledon cell number, which is fixed at the end of the mitotic phase, determines the final capacity to accumulate dry matter within legume seeds (i.e. seed size), which is ultimately correlated to crop yield within an agricultural setting (Lemontey *et al*., 2000).

## Competing interests

None declared.

## Author contributions

BMO designed the research. BMO, SH, VK and CR performed the research. CR and AHM provided mass spectrometry analysis. EN provided abscisic acid analysis. BMO and AHM wrote the paper with contributions from all authors.

## Disclaimer

The New Phytologist Foundation remains neutral with regard to jurisdictional claims in maps and in any institutional affiliations.

## Supporting information


**Fig. S1** Replicate immunoblots for data presented in Fig. 1.
**Fig. S2** Nutrient‐TOR signaling in Arabidopsis seedling roots and shoots.
**Fig. S3** Replicate immunoblots for data presented in Fig. 2.
**Fig. S4** The relative abundance of ^15^N‐labeled and unlabeled metabolites in Arabidopsis leaf discs following 6 h incubations in the light.
**Fig. S5** The relative abundance of ^15^N‐labeled and unlabeled metabolites in Arabidopsis leaf discs following 6 h incubations in the dark.
**Fig. S6** Immunoblots of RPS6‐Ser^240^ phosphorylation status following pea embryo incubations.
**Fig. S7** Developmental accumulation of starch, sucrose, Gln and protein in pea embryos.
**Fig. S8** Analysis of TOR‐dependent phosphorylation sites in developing pea embryos.
**Fig. S9** The FW changes of pea embryos in culture for 48 h.
**Fig. S10** Gene ontology analysis of differentially expressed transcripts in developing pea embryos incubated with or without AZD.

**Table S1** Arabidopsis leaf metabolomic analysis.
**Table S2** Pea embryo phosphoproteomics analysis. List of all identified phosphosites.
**Table S3** Pea embryo phosphoproteomics analysis. List of significantly different phosphosites between Gln and either AZD or Gln + AZD treatments.
**Table S4** Pea embryo phosphoproteomics analysis. Gene ontology analysis of significantly different TOR‐dependent phosphosites.
**Table S5** List of significantly different transcript abundancies identified from RNA sequencing analysis of pea embryos incubated in the presence and absence of AZD.
**Table S6** List of seed storage protein transcript levels quantified by RNA sequencing following pea embryo treatment in the presence and absence of AZD for 4 h.
Please note: Wiley is not responsible for the content or functionality of any Supporting Information supplied by the authors. Any queries (other than missing material) should be directed to the *New Phytologist* Central Office.

## Data Availability

The data that support the findings of this study are available in the Supporting Information of this article (Figs S1–S10; Tables S1–S6). Raw sequencing data were deposited at the NCBI Sequence Read Archive under project number PRJNA1251708.

## References

[nph70622-bib-0001] Belda‐Palazon B , Adamo M , Valerio C , Ferreira LJ , Confraria A , Reis‐Barata D , Rodrigues A , Meyer C , Rodriguez PL , Baena‐Gonzalez E . 2020. A dual function of SnRK2 kinases in the regulation of SnRK1 and plant growth. Nature Plants 6: 1345–1353. 33077877 10.1038/s41477-020-00778-w

[nph70622-bib-0002] Belda‐Palazon B , Costa M , Beeckman T , Rolland F , Baena‐Gonzalez E . 2022. ABA represses TOR and root meristem activity through nuclear exit of the SnRK1 kinase. Proceedings of the National Academy of Sciences, USA 119: e2204862119. 10.1073/pnas.2204862119 PMC9282376 35787039

[nph70622-bib-0003] Bernfeld E , Menon D , Vaghela V , Zerin I , Faruque P , Frias MA , Foster DA . 2018. Phospholipase D‐dependent mTOR complex 1 (mTORC1) activation by glutamine. The Journal of Biological Chemistry 293: 16390–16401. 30194281 10.1074/jbc.RA118.004972 PMC6200938

[nph70622-bib-0004] Busche M , Scarpin MR , Hnasko R , Brunkard JO . 2021. TOR coordinates nucleotide availability with ribosome biogenesis in plants. Plant Cell 33: 1615–1632. 33793860 10.1093/plcell/koab043 PMC8254494

[nph70622-bib-0005] Caldana C , Li Y , Leisse A , Zhang Y , Bartholomaeus L , Fernie AR , Willmitzer L , Giavalisco P . 2013. Systemic analysis of inducible target of rapamycin mutants reveal a general metabolic switch controlling growth in Arabidopsis thaliana. The Plant Journal 73: 897–909. 23173928 10.1111/tpj.12080

[nph70622-bib-0006] Cao P , Kim SJ , Xing A , Schenck CA , Liu L , Jiang N , Wang J , Last RL , Brandizzi F . 2019. Homeostasis of branched‐chain amino acids is critical for the activity of TOR signaling in Arabidopsis. eLife 8: e50747. 31808741 10.7554/eLife.50747 PMC6937141

[nph70622-bib-0007] Chellamuthu VR , Ermilova E , Lapina T , Lüddecke J , Minaeva E , Herrmann C , Hartmann MD , Forchhammer K . 2014. A widespread glutamine‐sensing mechanism in the plant kingdom. Cell 159: 1188–1199. 25416954 10.1016/j.cell.2014.10.015

[nph70622-bib-0008] Chopin F , Orsel M , Dorbe M‐F , Chardon F , Truong H‐N , Miller AJ , Krapp A , Daniel‐Vedele F . 2007. The Arabidopsis ATNRT2.7 nitrate transporter controls nitrate content in seeds. Plant Cell 19: 1590–1602. 17540716 10.1105/tpc.107.050542 PMC1913726

[nph70622-bib-0009] Christine R , Boutin J‐P . 1991. Metabolism of phloem‐borne amino acids in maternal tissues of fruit of nodulated or nitrate‐fed pea plants (*Pisum sativum* L). Journal of Experimental Botany 42: 207–214.

[nph70622-bib-0010] Crooks GE , Hon G , Chandonia JM , Brenner SE . 2004. WebLogo: a sequence logo generator. Genome Research 14: 1188–1190. 15173120 10.1101/gr.849004 PMC419797

[nph70622-bib-0011] Delgado LD , Nunez‐Pascual V , Riveras E , Ruffel S , Gutiérrez RA . 2024. Recent advances in local and systemic nitrate signaling in *Arabidopsis thaliana* . Current Opinion in Plant Biology 81: 102605. 39033715 10.1016/j.pbi.2024.102605

[nph70622-bib-0012] Deprost D , Yao L , Sormani R , Moreau M , Leterreux G , Nicolai M , Bedu M , Robaglia C , Meyer C . 2007. The Arabidopsis TOR kinase links plant growth, yield, stress resistance and mRNA translation. EMBO Reports 8: 864–870. 17721444 10.1038/sj.embor.7401043 PMC1973950

[nph70622-bib-0013] Dobin A , Davis CA , Schlesinger F , Drenkow J , Zaleski C , Jha S , Batut P , Chaisson M , Gingeras TR . 2012. Star: ultrafast universal RNA‐seq aligner. Bioinformatics 29: 15–21. 23104886 10.1093/bioinformatics/bts635 PMC3530905

[nph70622-bib-0014] Dobrenel T , Mancera‐Martinez E , Forzani C , Azzopardi M , Davanture M , Moreau M , Schepetilnikov M , Chicher J , Langella O , Zivy M *et al*. 2016. The Arabidopsis TOR kinase specifically regulates the expression of nuclear genes coding for plastidic ribosomal proteins and the phosphorylation of the cytosolic ribosomal protein S6. Frontiers in Plant Science 7: 1611. 27877176 10.3389/fpls.2016.01611 PMC5100631

[nph70622-bib-0015] Duran RV , Oppliger W , Robitaille AM , Heiserich L , Skendaj R , Gottlieb E , Hall MN . 2012. Glutaminolysis activates Rag‐mTORC1 signaling. Molecular Cell 47: 349–358. 22749528 10.1016/j.molcel.2012.05.043

[nph70622-bib-0016] Enganti R , Cho SK , Toperzer JD , Urquidi‐Camacho RA , Cakir OS , Ray AP , Abraham PE , Hettich RL , von Arnim AG . 2018. Phosphorylation of ribosomal protein RPS6 integrates light signals and circadian clock signals. Frontiers in Plant Science 8: 2210. 29403507 10.3389/fpls.2017.02210 PMC5780430

[nph70622-bib-0017] Fernandes SA , Angelidaki D‐D , Nüchel J , Pan J , Gollwitzer P , Elkis Y , Artoni F , Wilhelm S , Kovacevic‐Sarmiento M , Demetriades C . 2024. Spatial and functional separation of mTORC1 signalling in response to different amino acid sources. Nature Cell Biology 26: 1918–1933. 39385049 10.1038/s41556-024-01523-7 PMC11567901

[nph70622-bib-0018] Finkelstein RR , Gampala SS , Rock CD . 2002. Abscisic acid signaling in seeds and seedlings. Plant Cell 14: S15–S45. 12045268 10.1105/tpc.010441 PMC151246

[nph70622-bib-0019] Forde BG , Lea PJ . 2007. Glutamate in plants: metabolism, regulation, and signalling. Journal of Experimental Botany 58: 2339–2358. 17578865 10.1093/jxb/erm121

[nph70622-bib-0020] Fritz C , Mueller C , Matt P , Feil R , Stitt M . 2006. Impact of the C‐N status on the amino acid profile in tobacco source leaves. Plant, Cell & Environment 29: 2055–2076. 10.1111/j.1365-3040.2006.01580.x 17081241

[nph70622-bib-0021] Fu L , Liu Y , Qin G , Wu P , Zi H , Xu Z , Zhao X , Wang Y , Li Y , Yang S *et al*. 2021. The TOR‐EIN2 axis mediates nuclear signalling to modulate plant growth. Nature 591: 288–292. 33658715 10.1038/s41586-021-03310-y

[nph70622-bib-0022] Garneau MG , Lu MZ , Grant J , Tegeder M . 2021. Role of source‐to‐sink transport of methionine in establishing seed protein quantity and quality in legumes. Plant Physiology 187: 2134–2155. 34618032 10.1093/plphys/kiab238 PMC8644406

[nph70622-bib-0023] Gwinn DM , Shackelford DB , Egan DF , Mihaylova MM , Mery A , Vasquez DS , Turk BE , Shaw RJ . 2008. AMPK phosphorylation of raptor mediates a metabolic checkpoint. Molecular Cell 30: 214–226. 18439900 10.1016/j.molcel.2008.03.003 PMC2674027

[nph70622-bib-0024] Humphrey SJ , Karayel O , James DE , Mann M . 2018. High‐throughput and high‐sensitivity phosphoproteomics with the EasyPhos platform. Nature Protocols 13: 1897–1916. 30190555 10.1038/s41596-018-0014-9

[nph70622-bib-0025] Hussain S , Suda H , Nguyen CH , Yan D , Toyota M , Yoshioka K , Nambara E . 2024. Calcium signaling triggers early high humidity responses in *Arabidopsis thaliana* . Proceedings of the National Academy of Sciences, USA 121: e2416270121. 10.1073/pnas.2416270121 PMC11665853 39661062

[nph70622-bib-0026] Ingargiola C , Jehanno I , Forzani C , Marmagne A , Broutin J , Clement G , Leprince AS , Meyer C . 2023. The Arabidopsis target of rapamycin kinase regulates ammonium assimilation and glutamine metabolism. Plant Physiology 192: 2943–2957. 37042394 10.1093/plphys/kiad216

[nph70622-bib-0027] Jamsheer KM , Jindal S , Sharma M , Awasthi P , S S , Sharma M , Mannully CT , Laxmi A . 2022. A negative feedback loop of TOR signaling balances growth and stress‐response trade‐offs in plants. Cell Reports 39: 110631. 35385724 10.1016/j.celrep.2022.110631

[nph70622-bib-0028] Jewell JL , Kim YC , Russell RC , Yu FX , Park HW , Plouffe SW , Tagliabracci VS , Guan KL . 2015. Differential regulation of mTORC1 by leucine and glutamine. Science 347: 194–198. 25567907 10.1126/science.1259472 PMC4384888

[nph70622-bib-0029] Jin E , Wang S , Chen D , Wang J‐P , Zeng Y , Sun R , Zhang H‐T . 2024. P4HA2 activates mTOR via hydroxylation and targeting P4HA2‐mTOR inhibits lung adenocarcinoma cell growth. Oncogene 43: 1813–1823. 38654109 10.1038/s41388-024-03032-1 PMC11164680

[nph70622-bib-0030] Kravchenko A , Citerne S , Jehanno I , Bersimbaev RI , Veit B , Meyer C , Leprince AS . 2015. Mutations in the Arabidopsis Lst8 and raptor genes encoding partners of the TOR complex, or inhibition of TOR activity decrease abscisic acid (ABA) synthesis. Biochemical and Biophysical Research Communications 467: 992–997. 26459592 10.1016/j.bbrc.2015.10.028

[nph70622-bib-0031] Kreplak J , Madoui M‐A , Cápal P , Novák P , Labadie K , Aubert G , Bayer PE , Gali KK , Syme RA , Main D *et al*. 2019. A reference genome for pea provides insight into legume genome evolution. Nature Genetics 51: 1411–1422. 31477930 10.1038/s41588-019-0480-1

[nph70622-bib-0032] Lanfermeijer FC , van Oene MA , Borstlap AC . 1992. Compartmental analysis of amino‐acid release from attached and detached pea seed coats. Planta 187: 75–82. 24177969 10.1007/BF00201626

[nph70622-bib-0033] Lee K‐T , Liao H‐S , Hsieh M‐H . 2023. Glutamine metabolism, sensing and signaling in plants. Plant and Cell Physiology 64: 1466–1481. 37243703 10.1093/pcp/pcad054

[nph70622-bib-0034] Lemontey C , Mousset‐Déclas C , Munier‐Jolain N , Boutin JP . 2000. Maternal genotype influences pea seed size by controlling both mitotic activity during early embryogenesis and final endoreduplication level/cotyledon cell size in mature seed. Journal of Experimental Botany 51: 167–175. 10938823 10.1093/jexbot/51.343.167

[nph70622-bib-0035] Li B , Dewey CN . 2011. RSEM: accurate transcript quantification from RNA‐Seq data with or without a reference genome. BMC Bioinformatics 12: 323. 21816040 10.1186/1471-2105-12-323 PMC3163565

[nph70622-bib-0036] Li W , Liu J , Li Z , Ye R , Chen W , Huang Y , Yuan Y , Zhang Y , Hu H , Zheng P *et al*. 2024. Mitigating growth‐stress tradeoffs via elevated TOR signaling in rice. Molecular Plant 17: 240–257. 38053337 10.1016/j.molp.2023.12.002 PMC11271712

[nph70622-bib-0037] Liu GY , Sabatini DM . 2020. mTOR at the nexus of nutrition, growth, ageing and disease. Nature Reviews. Molecular Cell Biology 21: 183–203. 31937935 10.1038/s41580-019-0199-y PMC7102936

[nph70622-bib-0038] Liu Y , Duan X , Zhao X , Ding W , Wang Y , Xiong Y . 2021. Diverse nitrogen signals activate convergent ROP2‐TOR signaling in Arabidopsis. Developmental Cell 56: 1283–1295. 33831352 10.1016/j.devcel.2021.03.022

[nph70622-bib-0039] Liu Y , Hu J , Duan X , Ding W , Xu M , Xiong Y . 2025. Target of rapamycin (TOR): a master regulator in plant growth, development, and stress responses. Annual Review of Plant Biology 76: 341–371. 10.1146/annurev-arplant-083123-050311 39952681

[nph70622-bib-0040] Love MI , Huber W , Anders S . 2014. Moderated estimation of fold change and dispersion for RNA‐seq data with DESeq2. Genome Biology 15: 550. 25516281 10.1186/s13059-014-0550-8 PMC4302049

[nph70622-bib-0041] Lutt N , Brunkard JO . 2022. Amino acid signaling for TOR in eukaryotes: sensors, transducers, and a sustainable agricultural fuTORe. Biomolecules 12: 387. 35327579 10.3390/biom12030387 PMC8945916

[nph70622-bib-0042] Mallen‐Ponce MJ , Quintero‐Moreno AM , Gamez‐Arcas S , Grossman AR , Perez‐Perez ME , Crespo JL . 2025. Dihydroxyacetone phosphate generated in the chloroplast mediates the activation of TOR by CO(2) and light. Science Advances 11: eadu1240. 40249806 10.1126/sciadv.adu1240 PMC12007574

[nph70622-bib-0043] Matt P , Geiger M , Walch‐Liu P , Engels C , Krapp A , Stitt M . 2001. The immediate cause of the diurnal changes of nitrogen metabolism in leaves of nitrate‐replete tobacco: a major imbalance between the rate of nitrate reduction and the rates of nitrate uptake and ammonium metabolism during the first part of the light period. Plant, Cell & Environment 24: 177–190.

[nph70622-bib-0044] McAdam EL , Meitzel T , Quittenden LJ , Davidson SE , Dalmais M , Bendahmane AI , Thompson R , Smith JJ , Nichols DS , Urquhart S *et al*. 2017. Evidence that auxin is required for normal seed size and starch synthesis in pea. New Phytologist 216: 193–204. 28748561 10.1111/nph.14690

[nph70622-bib-0045] Meitzel T , Radchuk R , McAdam EL , Thormählen I , Feil R , Munz E , Hilo A , Geigenberger P , Ross JJ , Lunn JE *et al*. 2021. Trehalose 6‐phosphate promotes seed filling by activating auxin biosynthesis. New Phytologist 229: 1553–1565. 32984971 10.1111/nph.16956

[nph70622-bib-0046] Menand B , Desnos T , Nussaume L , Berger F , Bouchez D , Meyer C , Robaglia C . 2002. Expression and disruption of the Arabidopsis TOR (target of rapamycin) gene. Proceedings of the National Academy of Sciences, USA 99: 6422–6427. 10.1073/pnas.092141899 PMC122964 11983923

[nph70622-bib-0047] Meng D , Yang Q , Wang H , Melick CH , Navlani R , Frank AR , Jewell JL . 2020. Glutamine and asparagine activate mTORC1 independently of Rag GTPases. The Journal of Biological Chemistry 295: 2890–2899. 32019866 10.1074/jbc.AC119.011578 PMC7062167

[nph70622-bib-0048] Meng Y , Zhang N , Li J , Shen X , Sheen J , Xiong Y . 2022. TOR kinase, a GPS in the complex nutrient and hormonal signaling networks to guide plant growth and development. Journal of Experimental Botany 73: 7041–7054. 35781569 10.1093/jxb/erac282 PMC9664236

[nph70622-bib-0049] Millard P , Delépine B , Guionnet M , Heuillet M , Bellvert F , Létisse F . 2019. IsoCor: isotope correction for high‐resolution MS labeling experiments. Bioinformatics 35: 4484–4487. 30903185 10.1093/bioinformatics/btz209

[nph70622-bib-0050] Millerd A , Spencer D , Dudman W , Stiller M . 1975. Growth of immature pea cotyledons in culture. Functional Plant Biology 2: 51–59.

[nph70622-bib-0051] Morales‐Herrera S , Paul MJ , Van Dijck P , Beeckman T . 2024. SnRK1/TOR/T6P: three musketeers guarding energy for root growth. Trends in Plant Science 29: 1066–1076. 38580543 10.1016/j.tplants.2024.03.006

[nph70622-bib-0052] Moreau M , Azzopardi M , Clement G , Dobrenel T , Marchive C , Renne C , Martin‐Magniette ML , Taconnat L , Renou JP , Robaglia C *et al*. 2012. Mutations in the Arabidopsis homolog of LST8/GbetaL, a partner of the target of rapamycin kinase, impair plant growth, flowering, and metabolic adaptation to long days. Plant Cell 24: 463–481. 22307851 10.1105/tpc.111.091306 PMC3315227

[nph70622-bib-0053] Mubeen U , Jüppner J , Alpers J , Hincha DK , Giavalisco P . 2018. Target of rapamycin inhibition in *Chlamydomonas reinhardtii* triggers *de nov*o amino acid synthesis by enhancing nitrogen assimilation. Plant Cell 30: 2240–2254. 30228127 10.1105/tpc.18.00159 PMC6241278

[nph70622-bib-0054] Nukarinen E , Nagele T , Pedrotti L , Wurzinger B , Mair A , Landgraf R , Bornke F , Hanson J , Teige M , Baena‐Gonzalez E *et al*. 2016. Quantitative phosphoproteomics reveals the role of the AMPK plant ortholog SnRK1 as a metabolic master regulator under energy deprivation. Scientific Reports 6: 31697. 27545962 10.1038/srep31697 PMC4992866

[nph70622-bib-0055] O'Leary BM , Lee CP , Atkin OK , Cheng R , Brown TB , Millar AH . 2017. Variation in leaf respiration rates at night correlates with carbohydrate and amino acid supply. Plant Physiology 174: 2261–2273. 28615345 10.1104/pp.17.00610 PMC5543967

[nph70622-bib-0056] O'Leary BM , Oh GGK , Lee CP , Millar AH . 2020. Metabolite regulatory interactions control plant respiratory metabolism via target of rapamycin (TOR) kinase activation. Plant Cell 32: 666–682. 31888967 10.1105/tpc.19.00157 PMC7054028

[nph70622-bib-0057] Oh GGK , Kumari V , Millar AH , O'Leary BM . 2023. Alternative oxidase 1a and 1d enable metabolic flexibility during Ala catabolism in Arabidopsis. Plant Physiology 192: 2958–2970. 37128995 10.1093/plphys/kiad233

[nph70622-bib-0058] Oliveira IC , Brears T , Knight TJ , Clark A , Coruzzi GM . 2002. Overexpression of cytosolic glutamine synthetase. Relation to nitrogen, light, and photorespiration. Plant Physiology 129: 1170–1180. 12114571 10.1104/pp.020013 PMC166511

[nph70622-bib-0059] Orozco JM , Krawczyk PA , Scaria SM , Cangelosi AL , Chan SH , Kunchok T , Lewis CA , Sabatini DM . 2020. Dihydroxyacetone phosphate signals glucose availability to mTORC1. Nature Metabolism 2: 893–901. 10.1038/s42255-020-0250-5 PMC7995735 32719541

[nph70622-bib-0060] Palaniyandi SA , Chung G , Kim SH , Yang SH . 2015. Molecular cloning and characterization of the ABA‐specific glucosyltransferase gene from bean (*Phaseolus vulgaris* L.). Journal of Plant Physiology 178: 1–9. 25747288 10.1016/j.jplph.2015.01.015

[nph70622-bib-0061] Panwar V , Singh A , Bhatt M , Tonk RK , Azizov S , Raza AS , Sengupta S , Kumar D , Garg M . 2023. Multifaceted role of mTOR (mammalian target of rapamycin) signaling pathway in human health and disease. Signal Transduction and Targeted Therapy 8: 375. 37779156 10.1038/s41392-023-01608-z PMC10543444

[nph70622-bib-0062] Pegler JL , Grof CP , Patrick JW . 2023. Sugar loading of crop seeds – a partnership of phloem, plasmodesmal and membrane transport. New Phytologist 239: 1584–1602. 37306002 10.1111/nph.19058

[nph70622-bib-0063] Rabeh K , Oubohssaine M , Hnini M . 2024. TOR in plants: multidimensional regulators of plant growth and signaling pathways. Journal of Plant Physiology 294: 154186. 38330538 10.1016/j.jplph.2024.154186

[nph70622-bib-0064] Radchuk R , Conrad U , Saalbach I , Giersberg M , Emery RJ , Küster H , Nunes‐Nesi A , Fernie AR , Weschke W , Weber H . 2010. Abscisic acid deficiency of developing pea embryos achieved by immunomodulation attenuates developmental phase transition and storage metabolism. The Plant Journal 64: 715–730. 21105920 10.1111/j.1365-313X.2010.04376.x

[nph70622-bib-0065] Radchuk R , Radchuk V , Weschke W , Borisjuk L , Weber H . 2006. Repressing the expression of the SUCROSE NONFERMENTING‐1‐RELATED PROTEIN KINASE gene in pea embryo causes pleiotropic defects of maturation similar to an abscisic acid‐insensitive phenotype. Plant Physiology 140: 263–278. 16361518 10.1104/pp.105.071167 PMC1326049

[nph70622-bib-0066] Ren M , Qiu S , Venglat P , Xiang D , Feng L , Selvaraj G , Datla R . 2011. Target of rapamycin regulates development and ribosomal RNA expression through kinase domain in Arabidopsis. Plant Physiology 155: 1367–1382. 21266656 10.1104/pp.110.169045 PMC3046592

[nph70622-bib-0067] Ren M , Venglat P , Qiu S , Feng L , Cao Y , Wang E , Xiang D , Wang J , Alexander D , Chalivendra S *et al*. 2012. Target of rapamycin signaling regulates metabolism, growth, and life span in Arabidopsis. Plant Cell 24: 4850–4874. 23275579 10.1105/tpc.112.107144 PMC3556962

[nph70622-bib-0068] Rolletschek H , Hosein F , Miranda M , Heim U , Gotz KP , Schlereth A , Borisjuk L , Saalbach I , Wobus U , Weber H . 2005a. Ectopic expression of an amino acid transporter (VfAAP1) in seeds of *Vicia narbonensis* and pea increases storage proteins. Plant Physiology 137: 1236–1249. 15793070 10.1104/pp.104.056523 PMC1088317

[nph70622-bib-0069] Rolletschek H , Radchuk R , Klukas C , Schreiber F , Wobus U , Borisjuk L . 2005b. Evidence of a key role for photosynthetic oxygen release in oil storage in developing soybean seeds. New Phytologist 167: 777–786. 16101914 10.1111/j.1469-8137.2005.01473.x

[nph70622-bib-0070] Salem MA , Li Y , Bajdzienko K , Fisahn J , Watanabe M , Hoefgen R , Schottler MA , Giavalisco P . 2018. RAPTOR controls developmental growth transitions by altering the hormonal and metabolic balance. Plant Physiology 177: 565–593. 29686055 10.1104/pp.17.01711 PMC6001337

[nph70622-bib-0071] Scarpin MR , Leiboff S , Brunkard JO . 2020. Parallel global profiling of plant TOR dynamics reveals a conserved role for LARP1 in translation. eLife 9: e58795. 33054972 10.7554/eLife.58795 PMC7584452

[nph70622-bib-0072] Schepetilnikov M , Makarian J , Srour O , Geldreich A , Yang Z , Chicher J , Hammann P , Ryabova LA . 2017. GTPase ROP2 binds and promotes activation of target of rapamycin, TOR, in response to auxin. EMBO Journal 36: 886–903. 28246118 10.15252/embj.201694816 PMC5376970

[nph70622-bib-0073] Schwender J , Shachar‐Hill Y , Ohlrogge JB . 2006. Mitochondrial metabolism in developing embryos of *Brassica napus* . Journal of Biological Chemistry 281: 34040–34047. 16971389 10.1074/jbc.M606266200

[nph70622-bib-0074] Shi L , Wu Y , Sheen J . 2018. TOR signaling in plants: conservation and innovation. Development 145: dev160887. 29986898 10.1242/dev.160887 PMC6053665

[nph70622-bib-0075] Smith AM , Zeeman SC . 2006. Quantification of starch in plant tissues. Nature Protocols 1: 1342–1345. 17406420 10.1038/nprot.2006.232

[nph70622-bib-0076] Spriggs KA , Bushell M , Willis AE . 2010. Translational regulation of gene expression during conditions of cell stress. Molecular Cell 40: 228–237. 20965418 10.1016/j.molcel.2010.09.028

[nph70622-bib-0077] Stracka D , Jozefczuk S , Rudroff F , Sauer U , Hall MN . 2014. Nitrogen source activates TOR (target of rapamycin) complex 1 via glutamine and independently of Gtr/Rag proteins. The Journal of Biological Chemistry 289: 25010–25020. 25063813 10.1074/jbc.M114.574335 PMC4155668

[nph70622-bib-0078] Tanigawa M , Maeda T . 2017. An *in vitro* TORC1 kinase assay that recapitulates the Gtr‐independent glutamine‐responsive TORC1 activation mechanism on yeast vacuoles. Molecular and Cellular Biology 37: e00075‐17. 28483912 10.1128/MCB.00075-17 PMC5492174

[nph70622-bib-0079] Tanigawa M , Maeda T , Isono E . 2024. FYVE1/FREE1 is involved in glutamine‐responsive TORC1 activation in plants. iScience 27: 110814. 39297172 10.1016/j.isci.2024.110814 PMC11409180

[nph70622-bib-0080] Tegeder M , Masclaux‐Daubresse C . 2018. Source and sink mechanisms of nitrogen transport and use. New Phytologist 217: 35–53. 29120059 10.1111/nph.14876

[nph70622-bib-0081] Thompson JF , Madison JT , Muenster A‐ME . 1977. *In vitro* culture of immature cotyledons of soya bean (*Glycine max* L. Merr.). Annals of Botany 41: 29–39.

[nph70622-bib-0082] Tyanova S , Temu T , Cox J . 2016a. The MaxQuant computational platform for mass spectrometry‐based shotgun proteomics. Nature Protocols 11: 2301–2319. 27809316 10.1038/nprot.2016.136

[nph70622-bib-0083] Tyanova S , Temu T , Sinitcyn P , Carlson A , Hein MY , Geiger T , Mann M , Cox J . 2016b. The Perseus computational platform for comprehensive analysis of (prote)omics data. Nature Methods 13: 731–740. 27348712 10.1038/nmeth.3901

[nph70622-bib-0084] Valvezan AJ , Manning BD . 2019. Molecular logic of mTORC1 signalling as a metabolic rheostat. Nature Metabolism 1: 321–333. 10.1038/s42255-019-0038-7 PMC12569966 32694720

[nph70622-bib-0085] Van Leene J , Eeckhout D , Gadeyne A , Matthijs C , Han C , De Winne N , Persiau G , Van De Slijke E , Persyn F , Mertens T *et al*. 2022. Mapping of the plant SnRK1 kinase signalling network reveals a key regulatory role for the class II T6P synthase‐like proteins. Nature Plants 8: 1245–1261. 36376753 10.1038/s41477-022-01269-w

[nph70622-bib-0086] Van Leene J , Han C , Gadeyne A , Eeckhout D , Matthijs C , Cannoot B , De Winne N , Persiau G , Van De Slijke E , Van de Cotte B *et al*. 2019. Capturing the phosphorylation and protein interaction landscape of the plant TOR kinase. Nature Plants 5: 316–327. 30833711 10.1038/s41477-019-0378-z

[nph70622-bib-0087] Wang P , Zhao Y , Li Z , Hsu CC , Liu X , Fu L , Hou YJ , Du Y , Xie S , Zhang C *et al*. 2018. Reciprocal regulation of the TOR kinase and ABA receptor balances plant growth and stress response. Molecular Cell 69: 100–112 e106. 29290610 10.1016/j.molcel.2017.12.002 PMC5772982

[nph70622-bib-0088] Weber H , Borisjuk L , Wobus U . 2005. Molecular physiology of legume seed development. Annual Review of Plant Biology 56: 253–279. 10.1146/annurev.arplant.56.032604.144201 15862096

[nph70622-bib-0089] Wolfson RL , Sabatini DM . 2017. The dawn of the age of amino acid sensors for the mTORC1 pathway. Cell Metabolism 26: 301–309. 28768171 10.1016/j.cmet.2017.07.001 PMC5560103

[nph70622-bib-0090] Wu T , Hu E , Xu S , Chen M , Guo P , Dai Z , Feng T , Zhou L , Tang W , Zhan L *et al*. 2021. clusterProfiler 4.0: a universal enrichment tool for interpreting omics data. The Innovation 2: 100141. 34557778 10.1016/j.xinn.2021.100141 PMC8454663

[nph70622-bib-0091] Xiong Y , McCormack M , Li L , Hall Q , Xiang C , Sheen J . 2013. Glucose‐TOR signalling reprograms the transcriptome and activates meristems. Nature 496: 181–186. 23542588 10.1038/nature12030 PMC4140196

[nph70622-bib-0092] Yoneyama T , Suzuki A . 2020. Light‐independent nitrogen assimilation in plant leaves: nitrate incorporation into glutamine, glutamate, aspartate, and asparagine traced by 15N. Plants 9: 1303. 33023108 10.3390/plants9101303 PMC7600499

[nph70622-bib-0093] Zhang L , Garneau MG , Majumdar R , Grant J , Tegeder M . 2015. Improvement of pea biomass and seed productivity by simultaneous increase of phloem and embryo loading with amino acids. The Plant Journal 81: 134–146. 25353986 10.1111/tpj.12716

[nph70622-bib-0094] Zinsmeister J , Lalanne D , Terrasson E , Chatelain E , Vandecasteele C , Vu BL , Dubois‐Laurent C , Geoffriau E , Signor CL , Dalmais M *et al*. 2016. ABI5 is a regulator of seed maturation and longevity in legumes. Plant Cell 28: 2735–2754. 27956585 10.1105/tpc.16.00470 PMC5155344

