# Supplementary material for: Target of rapamycin signaling in pea embryos is dependent on glutamine but detached from seed storage protein biosynthesis
Source: New Phytol. 2025 Oct 15;248(6):2833–49. doi: 10.1111/nph.70622 (PMC12630444; doi:10.1111/nph.70622)
Supplement: Supplementary file 1 — Fig. S1 Replicate immunoblots for data presented in Fig. 1. Fig. S2 Nutrient‐TOR signaling in Arabidopsis seedling roots and shoots. Fig. S3 Replicate immunoblots for data presented in Fig. 2. Fig. S4 The relative abundance of 15N‐labeled and unlabeled metabolites in Arabidopsis leaf discs following 6 h incubations in the light. Fig. S5 The relative abundance of 15N‐labeled and unlabeled metabolites in Arabidopsis leaf discs following 6 h incubations in the dark. Fig. S6 Immunoblots of RPS6‐Ser240 phosphorylation status following pea embryo incubations. Fig. S7 Developmental accumulation of starch, sucrose, Gln and protein in pea embryos. Fig. S8 Analysis of TOR‐dependent phosphorylation sites in developing pea embryos. Fig. S9 The FW changes of pea embryos in culture for 48 h. Fig. S10 Gene ontology analysis of differentially expressed transcripts in developing pea embryos incubated with or without AZD. [file NPH-248-2833-s001.pdf]

***New Phytologist* Supporting Information**

Article title: **Target of Rapamycin (TOR) Signalling in Pea Embryos Is Dependent on Glutamine but Detached from Seed Storage Protein Biosynthesis**

Authors: Brendan M. O'Leary†, Suvi Honkanen, Vinti Kumari, Christoph Rampitsch, Eiji Nambara, A. Harvey Millar

Article acceptance date: 15 September 2025

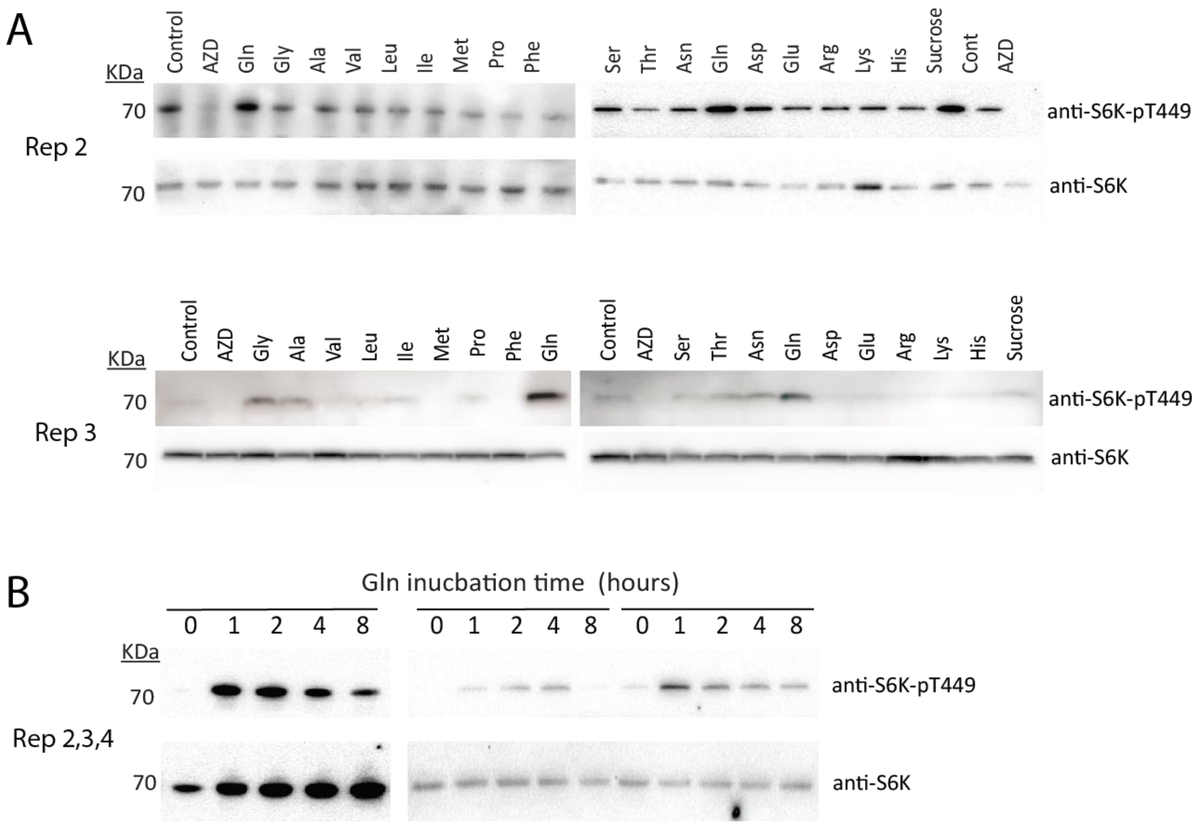


**Supplemental Figure 1:** Replicate immunoblots for data presented in Figure 1.
A) *S6K-HA* leaf discs were incubated 4 h in the present or absence of amino acids (10 mM), sucrose (20 mM) or AZD (2 µM). Protein extracts were subjected to urea-SDS-PAGE followed by immunoblotting with anti-S6K-pT449 or anti-S6K.
B) Timecourse incubation of *S6K-HA* leaf discs in Gln followed by urea-SDS-PAGE and immunoblotting analysis of S6K phosphorylation. Three replicates shown.

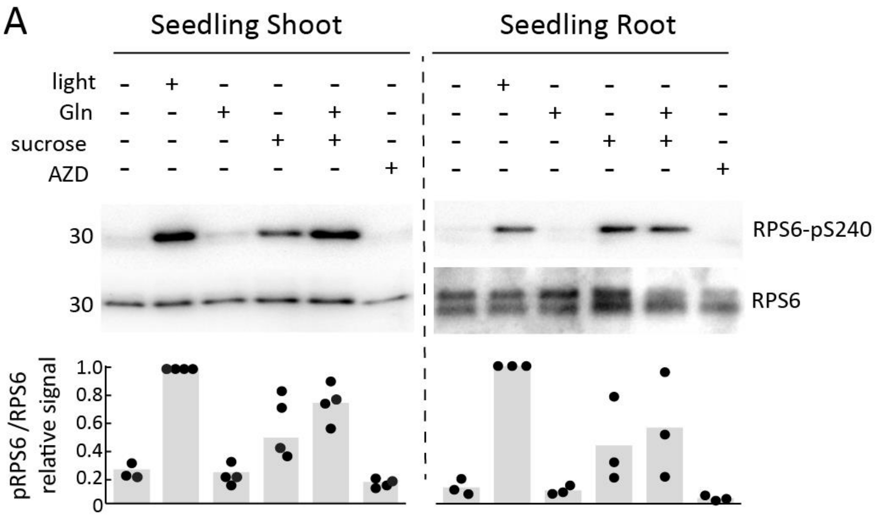


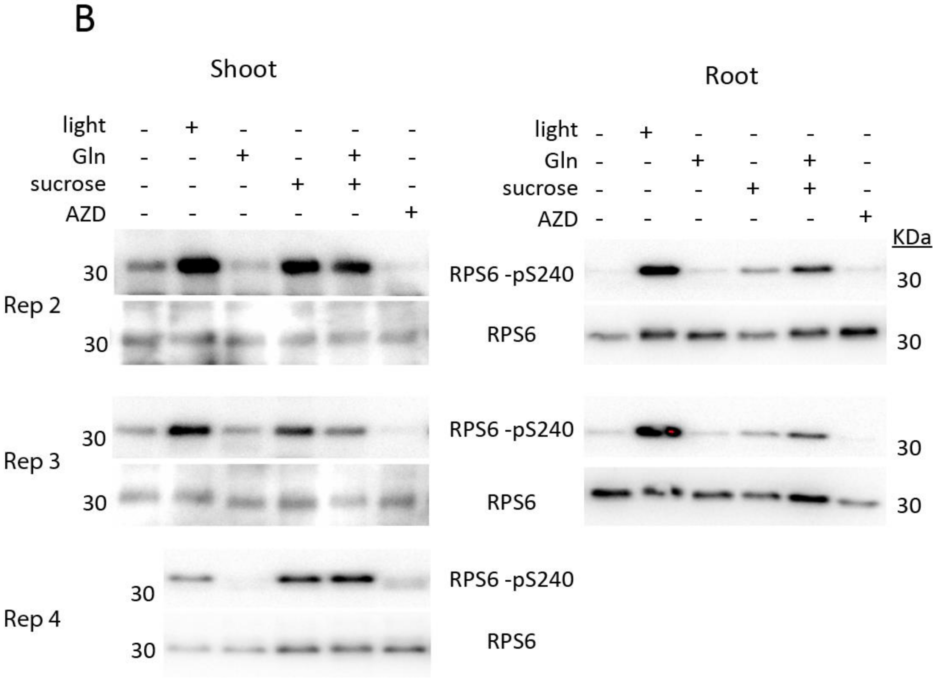


**Supplemental Figure 2:**

A) Arabidopsis seedlings were incubated in the dark for 16 hours followed by 4-hour incubations subject to light, Gln (10mM), sucrose (100 mM) or AZD (2 μM) treatments. Separate assessments of RPS6-Ser$^{240}$ phosphorylation status in separated root and shoot tissues were conducted (n=3-4).

B) Replicate root and shoot immunoblots.

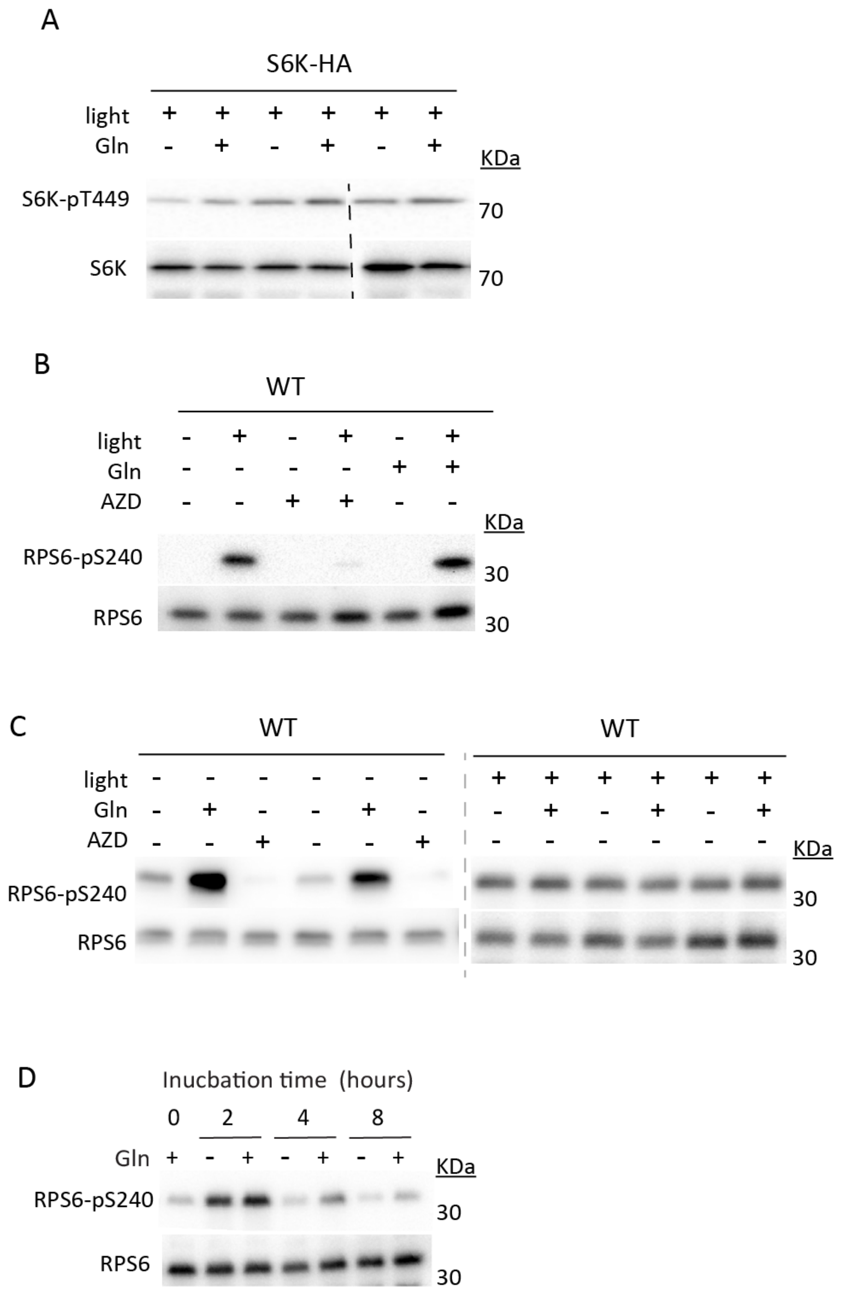


**Supplemental Figure 3:** Replicate immunoblots for data presented in Figure 2.
A) Replicate measurements of Gln's effect on S6K phosphorylation in leaf discs following 4 h incubation in the light. Supports Figure 2A-B.
B) Replicate immunoblot of RPS6-Ser240 phosphorylation in leaf discs following 4 h treatments with light or Gln. Supports Figure 2C.
C) Replicate measurements of Gln induction of RPS6-Ser240 phosphorylation in the light and the dark. Supports Figure 2D. Light and dark treatments blots were necessarily developed separately due to much higher signal intensity in light and are therefore not comparable.
D) Replicate timecourse measurements of RPS6-Ser240 phosphorylation following incubation of leaf discs with or without Gln at night. Supports Figure 2E.

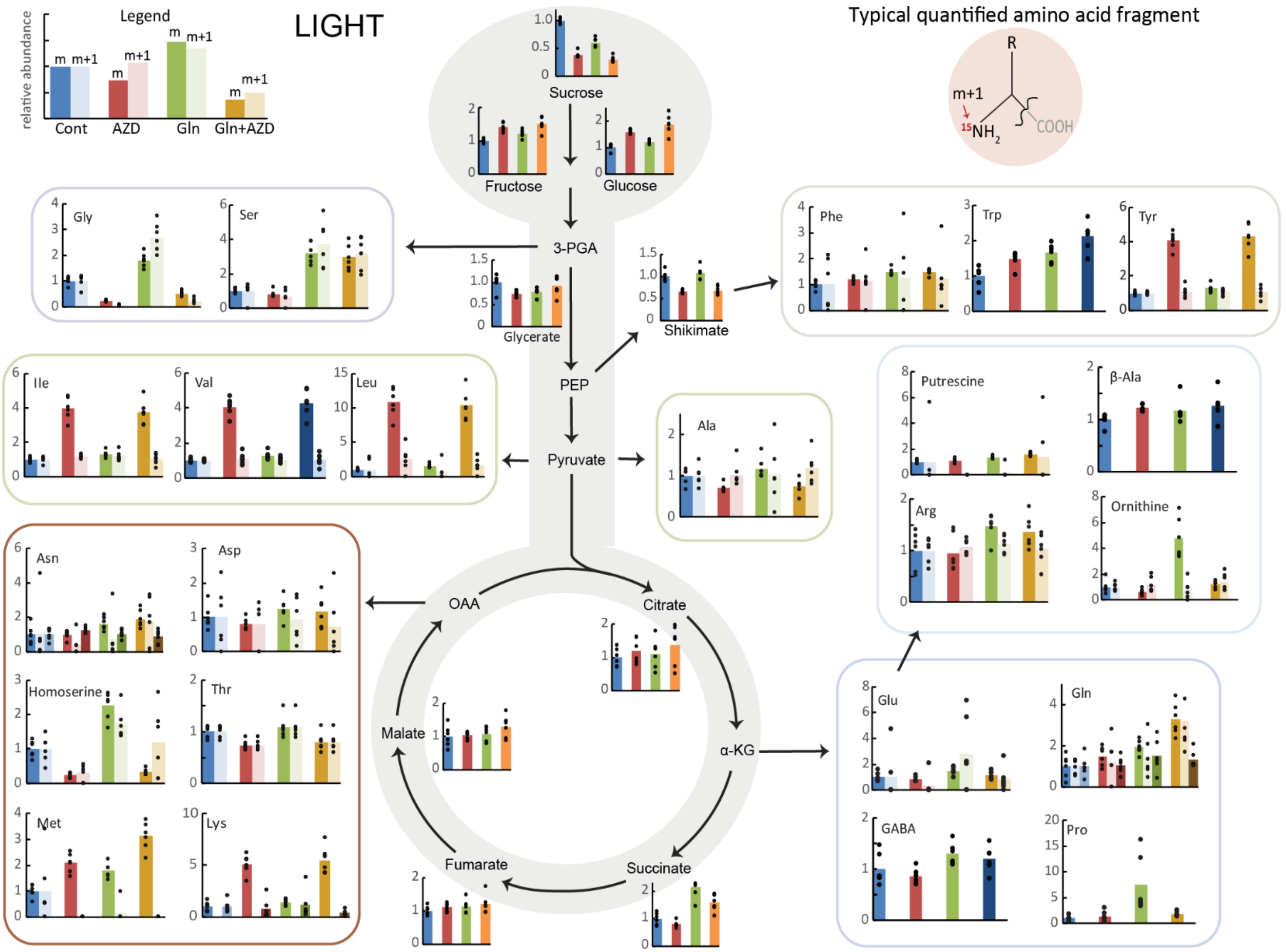


**Supplemental Figure 4:** The relative abundance of $^{15}N$ labelled and unlabelled metabolites in leaf discs following 6 h incubations in the light in 10 mM $^{15}NH_4{}^{15}NO_3$ with or without 2 µm AZD or 10 mM Gln. All values are expressed relative to control levels. Gln, Lys and Asn were also quantified for their M+2 signals. Amino acid M+1 signals were omitted for GABA, Pro Trp, β-Ala and Lys as they were below a reliable detection limit.

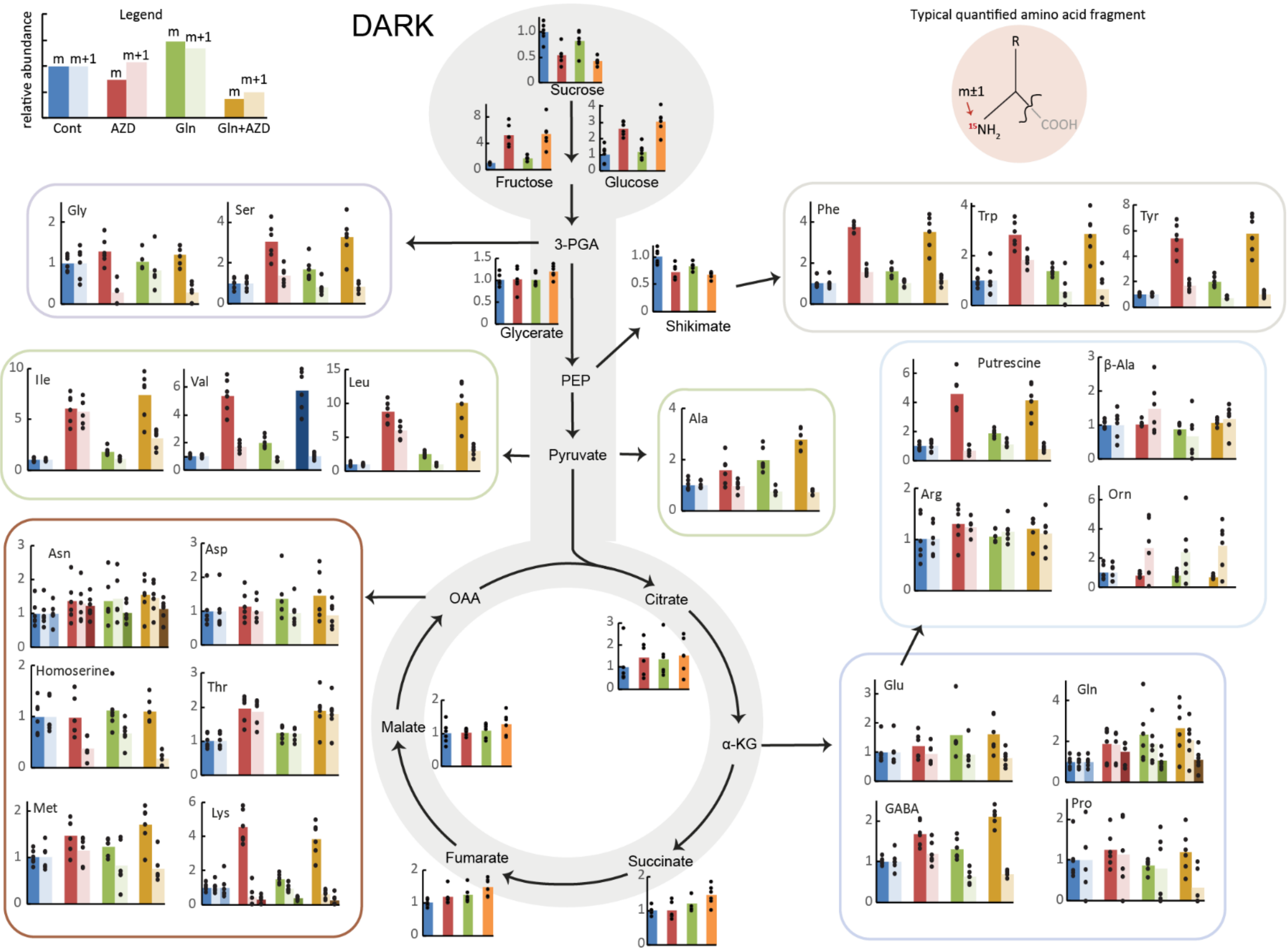


**Supplemental Figure 5**: The relative abundance of $^{15}N$ labelled and unlabelled metabolites in leaf discs following 6 h incubations in the dark in 10 mM $^{15}NH_4{}^{15}NO_3$ with or without 2 µm AZD and 10 mM Gln. All values are expressed relative to control levels (n=6). Gln, Lys and Asn were also quantified for their M+2 signals.

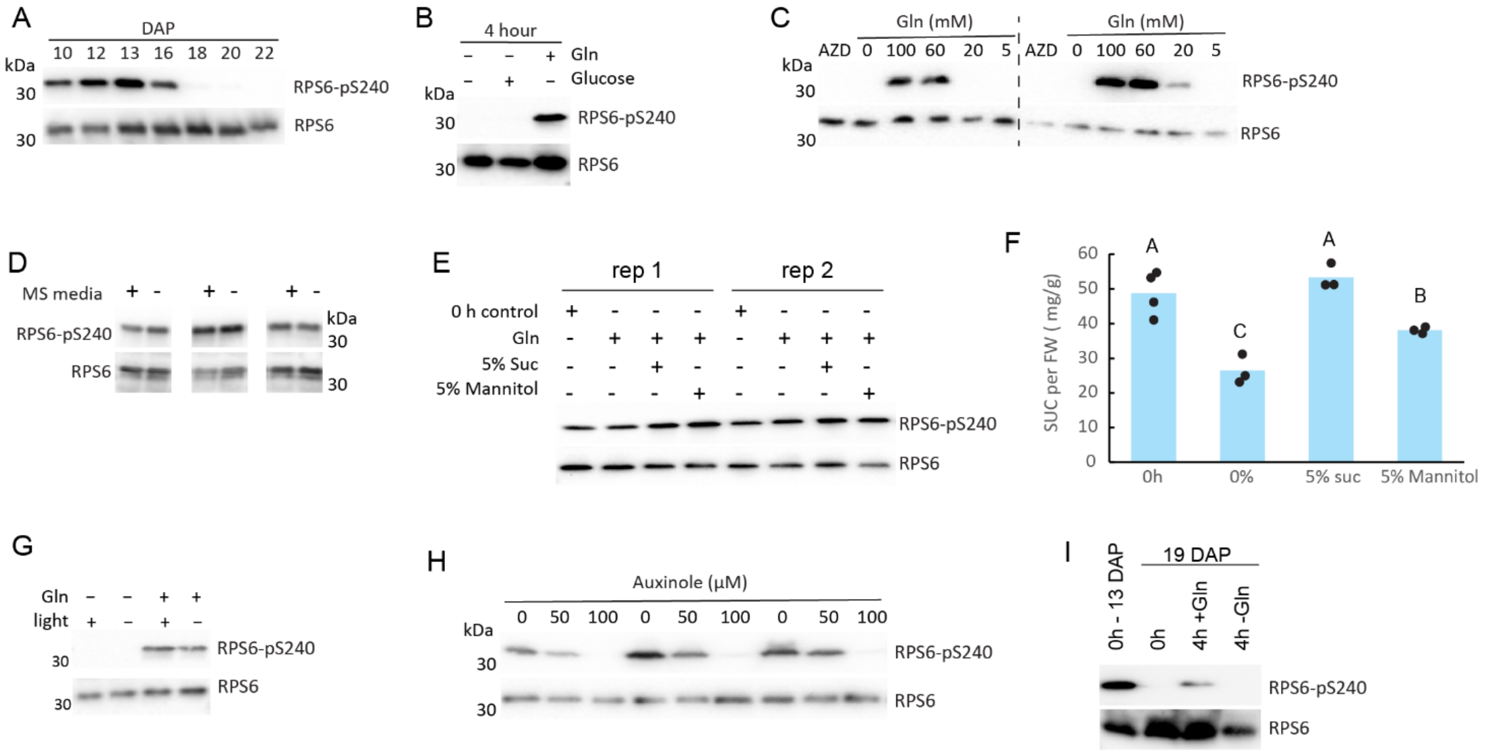


**Supplemental Figure 6:** Replicate immunoblots of RPS6-Ser$^{240}$ phosphorylation status following pea embryo incubations.

A) The *in vivo* RPS6-Ser$^{240}$ phosphorylation status in pea embryos throughout development, measured as days after pollination (DAP). Supports Figure 5A.

B) 4 h incubations with Gln (62.5 mM) or Glucose (100 mM). Supports Figure 5D.

C) 4h incubations with various concentrations of Gln or 2 µM AZD. Supports Figure 6A

D) Pea embryos were incubated for 4 h with Gln (62.5 mM) and sucrose (5% w/v) in the presence or absence of modified, nitrogen free MS salts. Three replicate experiments are shown.

E-F) To test the effect of osmotic pressure, pea embryos were incubated for 4 h with Gln (62.5 mM) in the presence and absence of (5% w/v) sucrose or mannitol. F) RPS6-Ser$^{240}$ phosphorylation status was measured. Two experimental replicates are shown. G) Sucrose content of embryos was measured following treatment. Letters indicate significant differences between groups (ANOVA, Tukey post-hoc testing; $p<0.05$).

G) Incubations with and without Gln in the light and dark. Supports Figure 6C.

H) The effect of auxinole on RPS6 phosphorylation in the presence of Gln and sucrose. Supports Figure 6D.

I) Incubation of older embryos at 19 DAP with and without Gln. Supports figure 6F

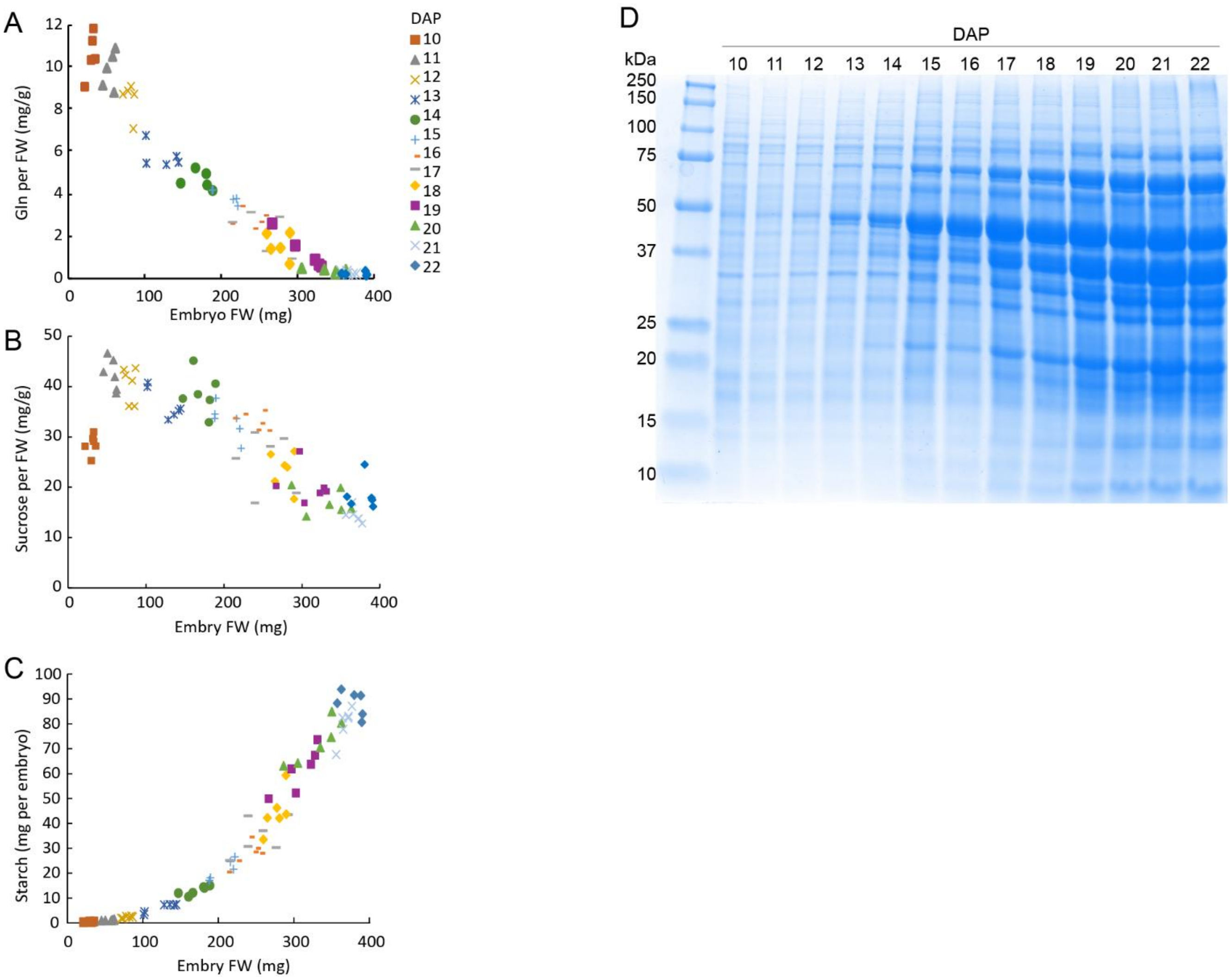


**Supplemental Figure 7:** Developmental accumulation of starch (A), sucrose (B), Gln (C) and protein (D) in pea embryos compared to FW and DAP. Seed storage proteins were visualized as prominent bands on a Coomassie stained SDS-PAGE gel.

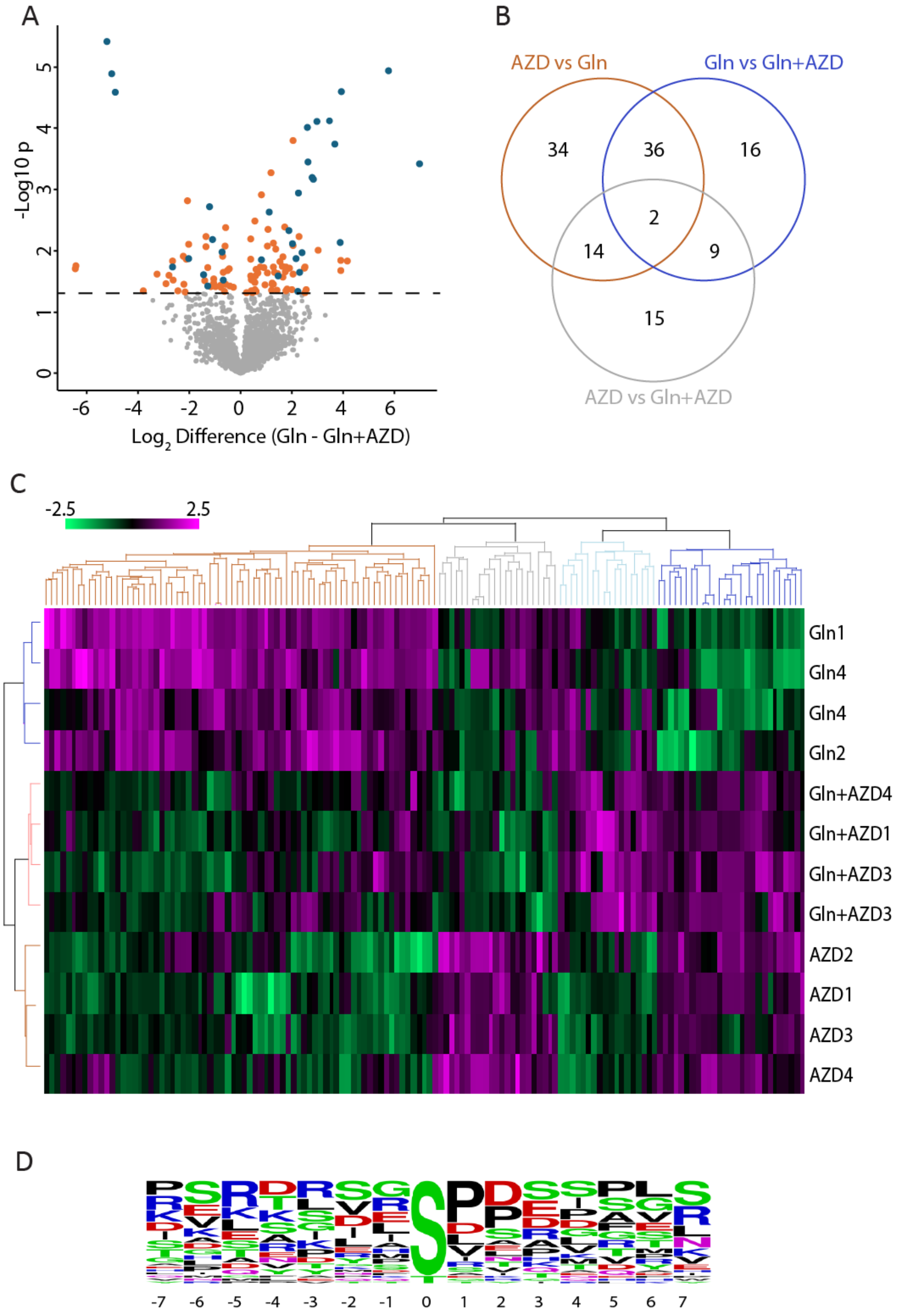


**Supplemental Figure 8**: Analysis of TOR-dependent phosphorylation sites in developing pea embryos.

A) Volcano plot showing the distribution of phosphorylation sites between Gln vs. Gln+AZD treatments. Significant differences that were also detected between Gln and AZD treatments are shown in blue (*p* <0.05; n=4)

B) Venn diagrams showing the distribution of significantly different phosphorylated residues among all treatments.

C) Hierarchical cluster analysis of the significantly different phosphorylation sites between all treatments.

D) Sequence motif analysis of TOR dependent phosphorylation sites.

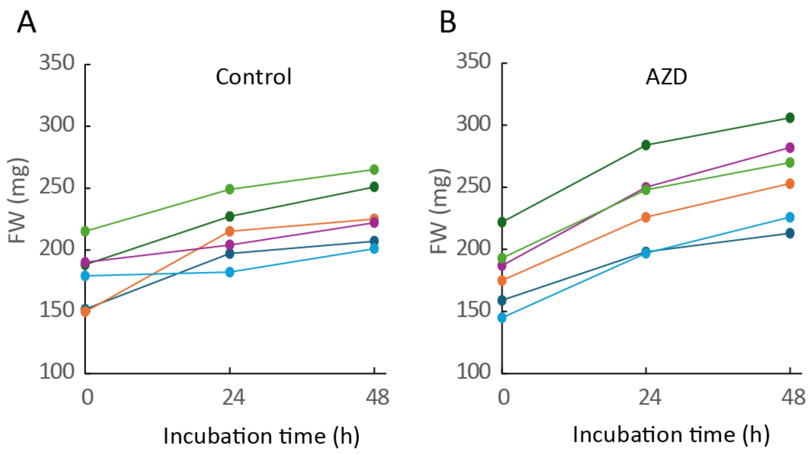


**Supplemental Figure 9:** The FW changes of individual pea embryos in culture for 48 hours with 10% sucrose and 62.5 mM Gln without (A) or with (B) the addition of 2 µm AZD.

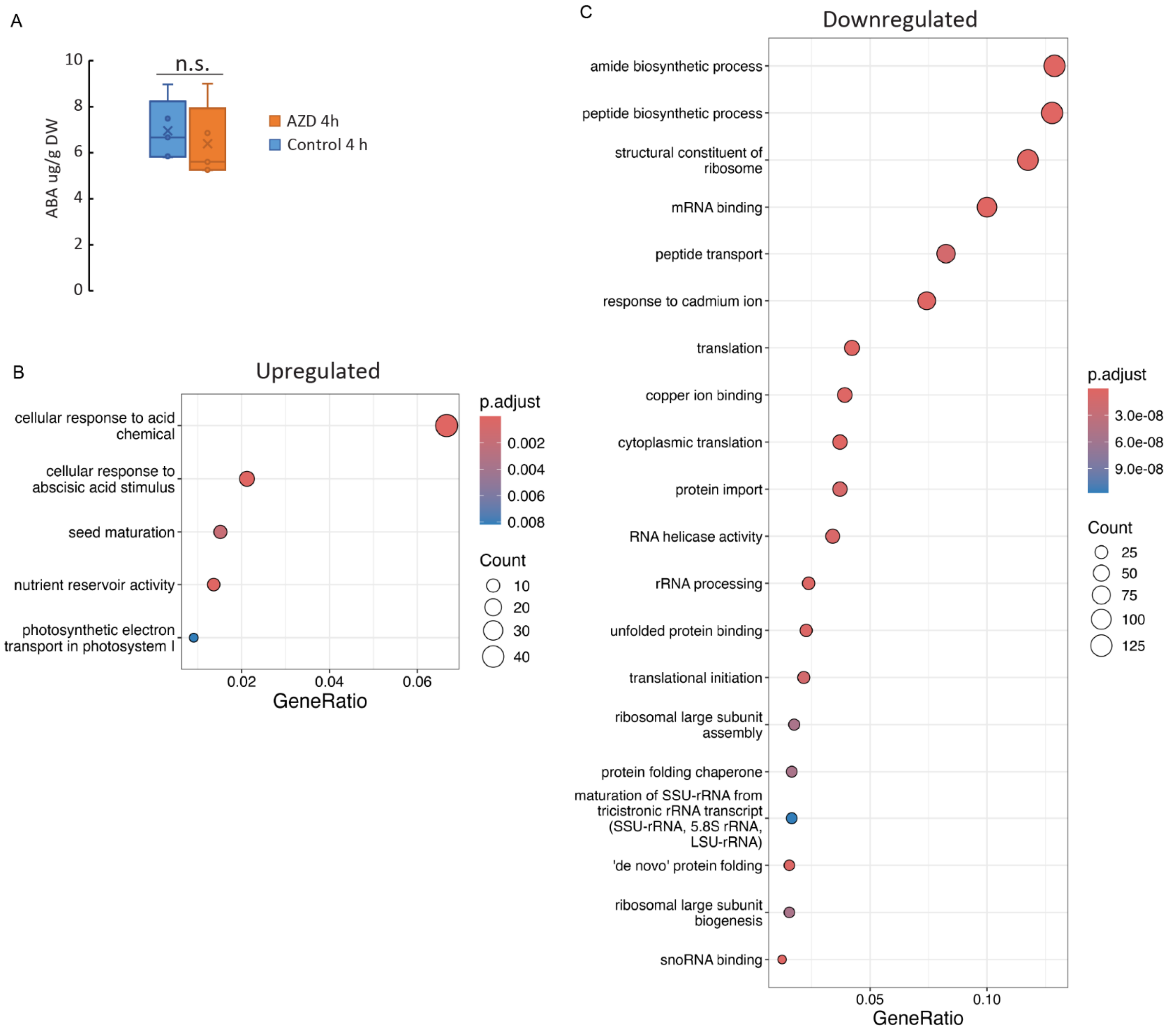


**Supplemental Figure 10:** Pea genes upregulated or downregulated in developing embryos incubated with Gln in the presence or absence of 2.5 µM AZD for 4 hours. A) ABA levels in control and AZD treated embryos not significantly different (*p*>0.05, t-test, n=5). Supports Figure 7G. B-C) Gene ontology enrichment analysis of significantly upregulated (B) and downregulated (C) genes ($Log_2$ fold change >0.4 or <-0.4, *p adj* <0.05) following transcriptomic analysis.
